# Dynamic landscape of microRNA expression in the feline small intestine during *Toxoplasma gondii* infection

**DOI:** 10.1186/s13071-026-07356-7

**Published:** 2026-04-11

**Authors:** Bintao Zhai, Bibo Bao, Shi-chen Xie, Hui Yang, Yang Liu, Weiwei Wang, Yaxin Zhou, Bing Li, Junjun He, Jiyu Zhang

**Affiliations:** 1 Lanzhou Institute of Husbandry and Pharmaceutical Sciences, Chinese Academy of Agricultural Sciences, Lanzhou, 730050 Gansu Province PR China; 2 Lanzhou Veterinary Research Institute, Chinese Academy of Agricultural Sciences, Lanzhou, 730046 Gansu Province PR China; 3 College of Medical, Qinghai University, Xining, 810016 Qinghai Province People’s Republic of China; 4 College of Life Science, Ningxia University, Yinchuan, 750021 Ningxia Hui Autonomous Region PR China; 5 College of Veterinary Medicine, Yunnan Agricultural University, Kunming, 650201 Yunnan Province PR China

**Keywords:** *Toxoplasmagondii*, Feline, MicroRNA, Small intestine, Host-pathogen interaction

## Abstract

**Background:**

*Toxoplasma gondii*, an obligate intracellular parasite with felids as its definitive hosts, undergoes sexual reproduction and oocyst shedding in the feline small intestine, a critical stage for its transmission. Small non-coding RNAs, particularly microRNAs (miRNAs), are crucial post-transcriptional regulators in host–pathogen interactions, but their role in the definitive host’s intestine during *T. gondii* infection remains unexplored.

**Methods:**

Fifteen cats were divided into control, primary infection (6, 10, 14 days post-infection, DPI), and secondary infection (SI) groups. Infection was confirmed via *B1* gene polymerase chain reaction (PCR). Small RNA sequencing was performed on the ileal epithelium. Bioinformatics analyses identified known and novel miRNAs, differential expression, target genes, and enriched pathways. Key miRNA–messenger RNA (mRNA) interactions were validated by dual-luciferase assay, and sequencing results were confirmed by quantitative PCR (qPCR).

**Results:**

Successful infection was molecularly confirmed. Sequencing identified 2666 miRNAs (2575 known, 91 novel). A dynamic pattern of differentially expressed (DE) miRNAs was observed, with peaks at 6 DPI (126), 10 DPI (122), 14DPI (36) and SI DPI (237), coinciding with active oocyst shedding. Key miRNAs like hsa-miR-199b-5p and ssc-miR-199b-5p were persistently downregulated. Target prediction and network analysis revealed complex interactions, including miR-199b-5p targeting *CYTH1* and *COQ7*. Functional enrichment highlighted significant involvement of target genes in the Rap1 and AMPK signaling pathways, as well as processes related to development and cellular organization. The novel_538–*CNN2* interaction was experimentally validated.

**Conclusions:**

This study provides the first comprehensive profile of miRNA expression in the feline small intestine during *T. gondii* infection. The temporal dynamics and specific dysregulation of miRNAs, coupled with enrichment in key pathways controlling cell adhesion and metabolism, suggest that *T. gondii* could orchestrate a sophisticated post-transcriptional program in its definitive host to potentially modify the intestinal environment for successful oocyst production and shedding. These findings lay the groundwork for future functional studies regarding the interplay between *T. gondii* and its definitive hosts.

**Graphical Abstract:**

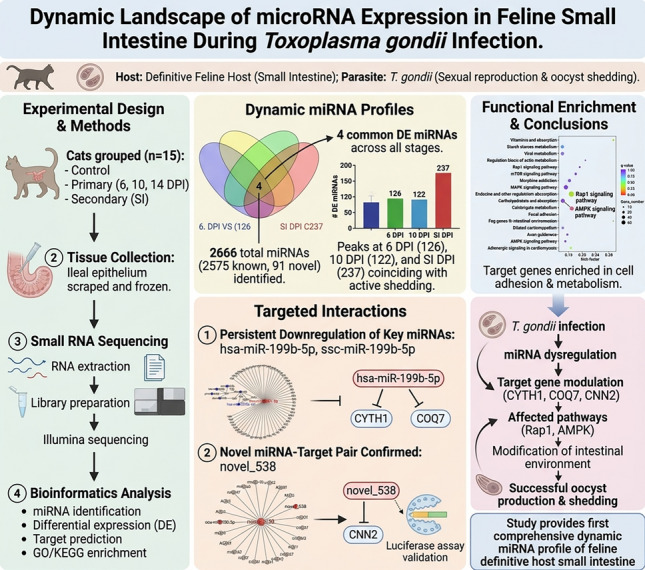

**Supplementary Information:**

The online version contains supplementary material available at 10.1186/s13071-026-07356-7.

## Background

*Toxoplasma gondii* (*T. gondii*) is an important obligate intracellular opportunistic pathogenic protozoan with a broad host range, capable of infecting various warm-blooded animals, including humans. Felids are currently its only known definitive hosts. The small intestine is the exclusive site for the sexual reproduction of *T. gondii*. After completing its sexual cycle within the intestinal epithelial cells of felids, the parasite sheds a vast number of oocysts, with reported estimates reaching up to hundreds of millions, into the environment [1]. These oocysts exhibit remarkable resilience and survival capability outdoors, making them a primary source of infection for intermediate hosts [2]. This characteristic may also be a major factor contributing to the genetic diversity and widespread prevalence of *T. gondii*. Surveys have indicated that the impact of toxoplasmosis on the livestock industry is closely related to the population and activity range of cats in farming areas [3]. In humans, accidental ingestion of food or water contaminated with *T. gondii* oocysts is one of the main routes of infection, which can lead to severe clinical symptoms or even death in infants, pregnant women, and immunocompromised individuals [4]. Therefore, the developmental process of *T. gondii* in the feline small intestine is a key factor in its extensive transmission.

RNA is one of the most fundamental biological molecules. Among its various forms, small non-coding RNAs (small RNAs) represent a class of endogenous regulatory factors with modulatory functions within cells, primarily including microRNAs (miRNAs) and small interfering RNAs (siRNAs). These small RNAs constitute non-protein regulatory networks that govern gene expression at the post-transcriptional level, participating in the regulation of cell proliferation, differentiation, and apoptosis, and organismal development [5]. miRNA, a member of the small RNA family, is approximately 20 nucleotides in length. It functions by binding to the 3′ untranslated region (UTR), coding region, or 5′ UTR of target messenger RNAs (mRNAs), thereby inhibiting their translation or promoting degradation, thus fulfilling its role in post-transcriptional gene regulation. Studies on the functions of miRNAs across multiple species have revealed that the gene regulatory roles played by miRNAs can assist parasitic protozoa in establishing infections within host cells and proliferating through mechanisms of immune evasion, which is crucial for the survival of these parasites [6–8]. Currently, research on *T. gondii* miRNAs remains relatively limited [6, 9–11]. *Toxoplasma gondii* can employ its unique physiological mechanisms to evade the host immune system, including the transfer of non-coding RNAs into host cells to modulate their functions [10, 12]. This is the first study to investigate the dynamic expression profiles of miRNAs in the feline small intestine following *T. gondii* infection. The findings of the study will contribute to a deeper understanding of the temporal dynamics of miRNA expression in the feline small intestine during *T. gondii* infection, providing a basis for further functional studies on oocyst production and shedding.

## Methods

### Parasite strain, infection, and sample collection

Fifteen domestic cats (*Felis catus*, Chinese Li Hua breed, 8 months old) were purchased from a local breeder and raised in a spacious cage at room temperature. Prior to purchase, relevant pathogen testing (enzyme-linked immunosorbent assay [ELISA]) was conducted on the cats to ensure the health and safety of the test animals. Subsequently, the 15 cats were randomly allocated into five groups: one control group, three primary infection groups (6_DPI, 10_DPI, 14_DPI), and one secondary infection group (SI_DPI), with three replicates in each group. Cats in the control group were administered 0.9% normal saline, whereas those in the *T. gondii*-infected groups were each challenged with 600 tissue cysts of the *T. gondii* PRU strain. Cats in the secondary infection group were re-challenged with the PRU strain 2 weeks (re-challenged at 27 DPI) after the cessation of oocyst shedding from the initially infected cats. All cats were humanely euthanized for tissue sample collection. Segments of the ileum from both the control and experimental groups were harvested at 6, 10, 14, and 27 DPI. The collected intestinal tissues were thoroughly rinsed with PBS to remove luminal contents. The intestinal epithelium was then carefully scraped using a cell lifter, immediately frozen in liquid nitrogen, and stored at −80 °C until further analysis. Other relevant operations and processes were performed as previously described [12].

### Polymerase chain reaction (PCR) detection of *Toxoplasma gondii* infection in small intestinal epithelium

In order to confirm the successful establishment of a *T. gondii* infection model in cats, fecal samples were collected daily and examined using the saturated sucrose flotation method. Additionally, genomic DNA was extracted from approximately 30 mg of small intestinal epithelium tissue using a commercial tissue genomic DNA extraction kit (TIANGEN, DP304-03, Tianjin, China) in accordance with the manufacturer’s instructions. To detect *T. gondii* infection, a PCR assay targeting the *B1* gene was performed as described previously [13].

### RNA isolation, library preparation, and small RNA sequencing (RNA-Seq)

Total RNA was extracted from the small intestinal epithelium tissue samples, followed by library preparation and sequencing. The specific operational methods and processes were performed as described previously [12]. For small RNA library construction, 3 μg of total RNA per sample was used as input. Libraries were prepared with the NEBNext^®^ Multiplex Small RNA Library Prep Set for Illumina^®^ (New England Biolabs [NEB], USA) according to the manufacturer’s protocol, with index codes incorporated for sample multiplexing. The procedure included ligation of the NEB 3′ SR adaptor specifically to the 3′ ends of miRNA, siRNA, and PIWI-associated RNA (piRNA). Following 3′ ligation, the SR RT primer was hybridized to any excess 3′ adaptor, converting it into double-stranded DNA to prevent adapter–dimer formation. Since double-stranded DNA is not a substrate for T4 RNA ligase 1, this also avoided ligation with the 5′ SR adaptor in the subsequent step. The 5′ ends of small RNAs were then ligated to the 5′ adapter, followed by first-strand complementary DNA (cDNA) synthesis using M-MuLV reverse transcriptase (RNase H^−^). PCR amplification was carried out with LongAmp Taq 2X Master Mix, SR primer for Illumina, and index primers. The resulting PCR products were size-selected on an 8% polyacrylamide gel (100 V, 80 min), and fragments of 140~160 base pairs (bp), corresponding to small RNAs with adapters, were excised and eluted in 8 μl of elution buffer. Finally, library quality was verified using the Agilent 2100 Bioanalyzer system with DNA high-sensitivity chips.

### Clustering, sequencing, and raw data processing

Following the manufacturer’s instructions, index-coded samples were clustered on a cBot cluster generation system using the TruSeq SR Cluster Kit v3-cBot-HS (Illumina). Subsequently, the library preparations were sequenced on an Illumina HiSeq 2500/2000 platform, generating 50-bp single-end reads.

The raw sequencing data in FASTQ format were first subjected to quality control. Custom Perl and Python scripts were employed to process the raw reads, yielding clean data by removing reads containing adapters, poly-N sequences, or low-quality bases. At the same time, Q20 and Q30 scores and GC content were calculated for the raw data. A specific length range of clean reads was selected for all subsequent analyses.

### Sequence alignment and miRNA identification

Clean small RNA tags were aligned to the reference genome using Bowtie without mismatch to analyze their expression and genomic distribution. Since there is no miRNA library for cats, we used a merged miRNA library approach for sequence alignment. For known miRNA identification, the aligned tags were compared against the miRBase 20.0 database using the mirdeep2 and sRNA-tools-cli software to identify known miRNAs and predict their secondary structures.

To filter out non-miRNA sequences, small RNA tags were mapped to Repbase, RepeatMasker, and the Rfam database to exclude those originating from protein-coding genes, repeats, ribosomal RNAs (rRNAs), transfer RNAs (tRNAs), small nuclear RNAs (snRNAs), and small nucleolar RNAs (snoRNAs). The remaining unannotated tags were used for novel miRNA prediction. miREvo and mirdeep2 software were integrated to identify novel miRNAs by assessing the characteristic hairpin structures of miRNA precursors, Dicer cleavage sites, and minimum free energy.

### Annotation, quantification, and differential expression

A comprehensive annotation summary was generated for all small RNA tags. Since a single tag could map to multiple categories, a priority rule (known miRNA > rRNA > tRNA > snRNA > snoRNA > repeat > gene > natural antisense [NAT]-siRNA > novel miRNA > trans-acting (ta)-siRNA) was applied to assign each unique small RNA to a single category. The proportion of total rRNA served as a sample quality indicator.

miRNA expression levels were quantified and normalized using transcripts per million (TPM). For samples with biological replicates, differential expression analysis between conditions was performed using the DESeq (differential expression analysis for sequence data) R package (v1.8.3). Adjusted *P*-values (Benjamini–Hochberg method) < 0.05 were considered statistically significant for differentially expressed (DE) miRNAs.

### Additional miRNA analyses

Further analyses included miRNA editing detection by allowing one mismatch in the seed region (positions 2–8) during alignment to mature miRNAs, as nucleotide changes here can alter target specificity. miRNA family analysis was conducted by comparing identified miRNAs against the miFam database (http://www.mirbase.org/ftp.shtml) for known miRNAs and submitting novel miRNA precursors to Rfam (http://rfam.sanger.ac.uk/search/). Finally, miRNA target genes were predicted using miRanda for animal species. Cytoscape 3.9.1 software was used to map the miRNA-gene interaction network.

### Dual-luciferase reporter gene assay

To investigate the interaction between miRNAs and their target mRNAs, two pairs of miRNA–mRNAs (novel_538–*CNN2*, hsa-miR-10395-3p–*RRP7A*) were randomly selected by dual-luciferase reporter gene assay. This study utilized the pmirGLO vector for restriction enzyme digestion and molecular cloning, with 293 T cells employed as the host system and NC-miRNA serving as the internal reference miRNA. The construction of pmirGLO-WT/MUT was synthesized by Sangon Biotech (https://store.sangon.com/userLogin, Shanghai, China). Cell transfection was performed following established literature protocols [14], and luciferase activity was detected according to the instructions provided in the dual-luciferase reporter assay kit (Vazyme, lot: DL101-01, Nanjing Nuoweizan Biotechnology Co., Ltd). All dual-luciferase reporter assays were performed in three or more independent biological replicates, each with three technical repeats, to ensure reproducibility and statistical robustness. Relative luciferase activity across groups was compared using a two-tailed *t*-test, with a value of *P* < 0.05 considered statistically significant.

### GO and KEGG enrichment analysis

Gene Ontology (GO) enrichment analysis was performed on the potential target genes of DE miRNAs using GOseq, which employs a Wallenius non-central hypergeometric distribution to correct for gene length bias. For pathway analysis, the Kyoto Encyclopedia of Genes and Genomes (KEGG) database (https://www.genome.jp/kegg/pathway.html) was utilized to interpret high-level functions of biological systems from molecular-level data, with a value of *P* < 0.05 considered indicative of significant enrichment.

### Verification of miRNA results by miRNA stem-loop fluorescence quantitative PCR

miRNA stem-loop fluorescence quantitative PCR (qPCR) was used to verify the miRNA results. The expression levels for 11 miRNAs were determined by qPCR using the same RNA samples that were used for the sequencing. Total RNA was extracted using the TRNzol Universal kit (TIANGEN, DP424, Beijing, China), and the miRcute Plus miRNA first-strand cDNA synthesis kit (TIANGEN, KR211-01, Beijing, China) was used to complete the A-tailing and reverse transcription of miRNA into cDNA. The expression of miRNA in each sample was detected by the miRcute Plus miRNA qPCR detection kit (TIANGEN, FP411-01, and Beijing, China). Relative miRNA expression was calculated using the 2^−ΔΔCt^ method, with snRNA U6 serving as the reference gene. All qPCR reactions were performed in triplicate, and the raw cycle threshold (Ct) values are provided in Table S1. This study adheres to the Minimum Information for Publication of Quantitative Real-Time PCR Experiments (MIQE) guidelines. The miRNA upstream primers (F) used in this study are shown in Table 1 and the downstream primers used in the experiment are universal downstream primers in the fluorescence quantitative kit. Eleven miRNAs were randomly selected for qPCR verification. All qPCR reactions were performed as described previously [12].

**Table 1:** Primers for microRNA stem-loop fluorescence quantitative PCR

Primer name	Primer sequence (5′ to 3′)
hsa-mir-451a-F	CTGCGCGAAAACCGTTACCATT
hsa-miR-199b-5p-F	CACTGCCTCCCAGTGTTTAGACT
ssc-mir-199b-F	CACTGCCTCCCAGTGTTTAGACT
mmu-mir-129b-F	AATCAATCAAGCCCAGACCGCA
chi-miR-451-5p-F	GCCGCGGAAACCGTTACCAT
novel_474-F	AACAGAGCCCCAAGTGGGACT
novel_538-F	AATCGGCGTCTTAGCTGAGCG
novel_279-F	AACACGCAAAGTGCTCCCATTTT
novel_442-F	CCTGCTGGTATGAATGGTGCCT
novel_370-F	ACGGCACTGTTGACTGTGTCC
novel_416-F	AAGCTGAGTGGGGCTGGAGAA
miRNA-R	ATCCAGTGCAGGGTCCGAGG
U6-F	CTCGCTTCGGCAGCACA
U6-R	AACGCTTCACGAATTTGCGT

Reverse primers (miRNA-R) are universal, and the reverse primers of different miRNAs are the same

## Results

### Successful establishment of *T. gondii* infection confirmed by PCR

**Fig. 1:**
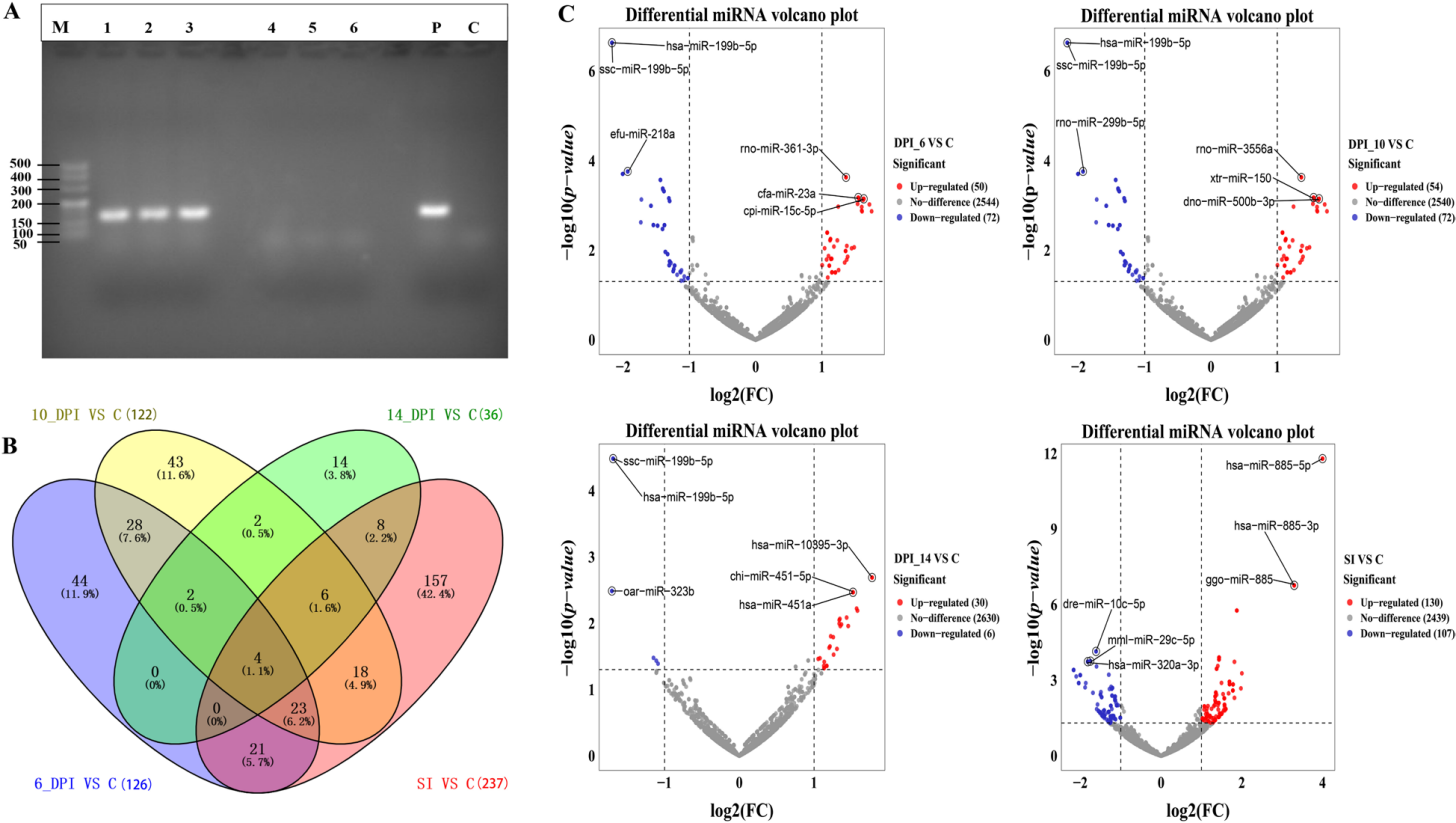
PCR detection of *Toxoplasma gondii* infection and the distribution of the differentially expressed (DE) miRNA. PCR detection of *T. gondii* infection in cat small intestine by *B1* gene (**A**). The order of the sample holes is as follows: M: Trans 500 plus DNA marker, random experimental group sample DNA (lanes 1–3), random control group sample DNA (lanes 4–6), P: *T. gondii* DNA positive control, N: negative standard product. The Venn diagram distribution between each infection group (**B**). Volcano of the up/downregulated DE miRNA (**C**). Red indicates upregulation, blue indicates downregulation, and gray indicates no significant difference. The top three significantly enriched upregulated and downregulated DE miRNA are marked with horizontal lines

*Toxoplasma gondii* infection was confirmed by PCR detection of the *B1* gene in the experimental group at 6, 10, and 14 DPI, while all control samples remained negative (Fig. 1A). Despite the absence of obvious clinical symptoms in both infected and control groups, the molecular data unequivocally established successful infection.

### Identification of known and novel miRNAs in feline intestinal epithelium

The clean reads (Table S1) were subsequently size-filtered and mapped to the reference sequence, followed by BLAST (Basic Local Alignment Search Tool) analysis against miRBase. This process led to the identification of 2666 miRNAs, comprising 2575 known miRNAs and 91 novel ones, distributed across 270 miRNA families (Table S2). As show in Figure S1, analysis of gene expression patterns and inter-sample correlations revealed highly similar expression profiles among samples, demonstrating the reliability of the experimental procedure and the appropriateness of sample selection.

### Temporal dynamics of differentially expressed miRNAs

In this study, differential expression of miRNAs was detected at four time points post-infection: 126 at 6 DPI, 122 at 10 DPI, 36 at 14 DPI, and 237 at SI DPI (Table S3). Four DE small RNAs (hsa-miR-199b-5p, ssc-miR-199b-5p, mmu-miR-129b-3p, mmu-miR-129-5p) were commonly identified across all four time points (Fig. 1B). Furthermore, analysis of miRNA expression in feline small intestine following *T. gondii* infection revealed five upregulated and 72 downregulated at 6 DPI; 54 upregulated and 72 downregulated at 10 DPI; 30 upregulated and six downregulated at 14 DPI; and 130 upregulated and 107 downregulated at SI DPI. At each time point, the top three most significantly upregulated and downregulated small RNAs were identified and labeled (Fig. 1C). These included ssc-miR-199b-5p, hsa-miR-199b-5p, oar-miR-323b, hsa-miR-10395-3p, chi-miR-451-5p, hsa-miR-451a, rno-miR-299b-5p, rno-miR-3556a, xtr-miR-150, dno-miR-500b-3p, efu-miR-218a, rno-miR-361-3p, cfa-miR-23a, cpi-miR-15c-5p, dre-miR-10c-5p, mml-miR-29c-5p, hsa-miR-320a-3p, hsa-miR-885-3p, ggo-miR-885, and hsa-miR-885-5p. Notably, both ssc-miR-199b-5p and hsa-miR-199b-5p were consistently labeled as downregulated at 6, 10, and 14 DPI.

### Clustering analysis reveals co-expression patterns of miRNAs

Samples with similar expression profiles were clustered together, as shown in Figure S2A. Interestingly, ssc-miR-199b-5p clusters with hsa-miR-199b-5p, mmu-miR-129b-5p pairs with mmu-miR-129b-3p, and chi-miR-451-5p groups with cpi-miR-451-5p.

Using K-means clustering, the DE miRNAs were successfully categorized into four subclusters (subcluster_1 to subcluster_4). Subcluster_2 contained the highest number of miRNAs, with the majority of significantly DE miRNAs grouped within this subcluster (Figure S2B).

Through SOM (self-organizing map) clustering, the miRNAs were divided into 29 subclusters. Among these, the significantly DE miRNAs were primarily clustered in subcluster_3_1 and subcluster_5_6 (Figure S2C).

Family analysis of the screened DE miRNAs revealed their distribution across 61 species, with *Mus musculus* and *Homo sapiens* comprising the majority. Additionally, nine novel miRNAs were categorized into a separate group (Figure S2D).

### Predicted miRNA–target interaction networks across infection stages

**Fig. 2:**
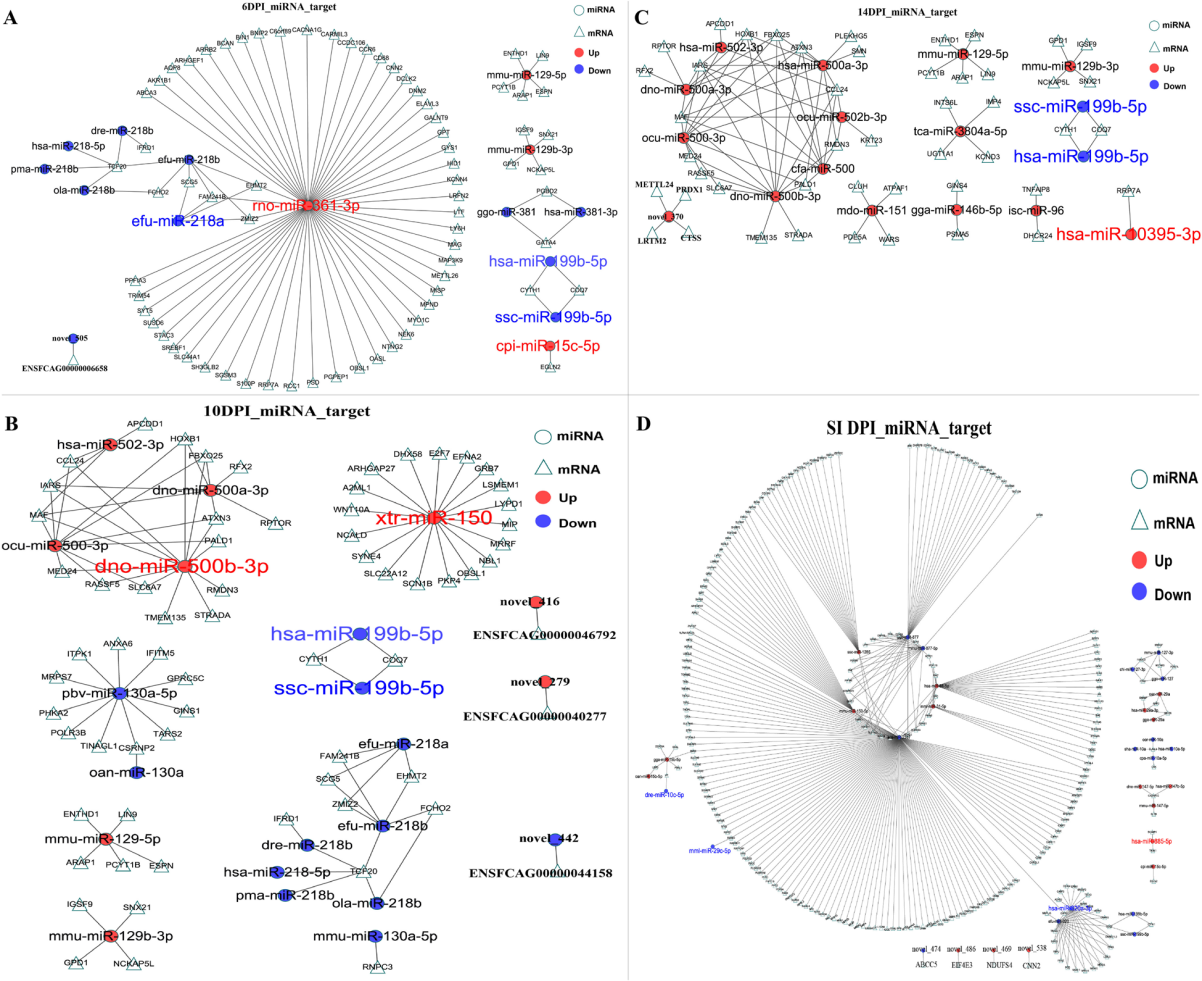
miRNA target gene prediction Target gene predictions for differentially expressed miRNAs are presented for each infection time point: 6 DPI (**A**), 10 DPI (**B**), 14 DPI (**C**), and SI DPI (**D**). In the interaction networks, circles represent miRNAs and triangles represent mRNAs. Upregulated miRNAs are highlighted in red, and downregulated ones in blue. Selected miRNAs corresponding to the top three most significantly differentially expressed miRNAs in the volcano plot are outlined with thicker borders in their respective colors

Potential target genes of DE miRNAs (including known and novel miRNAs) were predicted by intersecting the results. At 6 DPI, 14 miRNAs were predicted to target 71 mRNAs (Fig. 2A, Table S2). rno-miR-361-3p targets 53 mRNAs. efu-miR-218a regulates four mRNAs and indirectly modulates efu-miR-218b through three of these target mRNAs. mmu-miR-129b-3p and mmu-miR-129-5p target four and five mRNAs, respectively. hsa-miR-199b-5p and ssc-miR-199b-5p mutually regulate each other via the mRNAs *CYTH1* and *COQ7*. cpi-miR-15c-5p targets the *EGLN2* gene, while novel_505 targets the gene ENSFCAG00000006658.

At 10 DPI, 18 miRNAs were predicted to target 64 mRNAs (Fig. 2B). We found that the upregulated xtr-miR-150 targets 18 mRNAs, and the upregulated dno-miR-500b-3p targets 13 mRNAs. The downregulated hsa-miR-199b-5p and ssc-miR-199b-5p mutually regulate each other via the mRNAs *CYTH1* and *COQ7*. In addition, the upregulated novel_16 targets the gene ENSFCAG00000046792, the upregulated novel_279 targets ENSFCAG00000040277, and the downregulated novel_442 targets ENSFCAG00000044158.

At 14 DPI, 43 target mRNAs were predicted for 16 miRNAs (Fig. 2C). Upregulated mmu-miR-129b-3p and mmu-miR-129-5p target four and five mRNAs, respectively. The downregulated hsa-miR-199b-5p and ssc-miR-199b-5p mutually regulate each other through the mRNAs *CYTH1* and *COQ7*. Furthermore, upregulated hsa-miR-10395-3p specifically targets the *RRP7A* gene, while upregulated novel_370 simultaneously targets four mRNAs.

Finally, at SI DPI, 30 miRNAs were predicted to target 246 mRNAs (Fig. 2D). Downregulated dre-miR-10c-5p interacts with upregulated gga-miR-15b-5p via the *VIT* gene, while downregulated mml-miR-29c-5p interacts with downregulated bta-miR-2387 through the *RBM20* gene. Upregulated hsa-miR-885-5p regulates *SCAMP5* and *TRAK1* genes, and downregulated hsa-miR-320a-3p targets 24 mRNAs. Furthermore, upregulated novel_538, novel_469, and novel_486 target *CNN2*, *NDUFS4*, and *EIF4E3*, respectively, whereas downregulated novel_474 regulates the *ABCC5* gene.

**Fig. 3:**
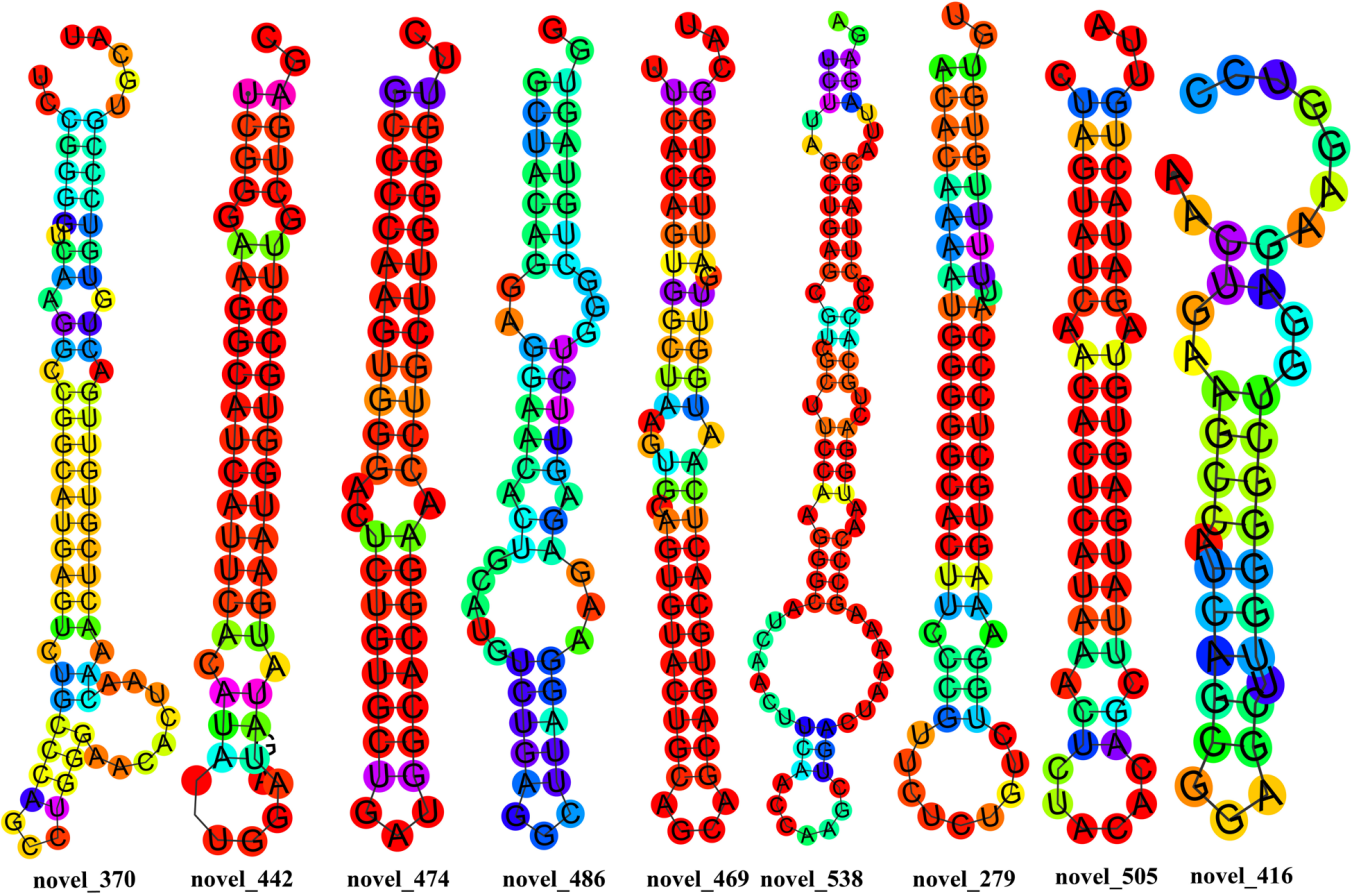
The novel miRNA sequence structural characteristics and positional entropy predicted by RNAfold

To further evaluate the biological plausibility of the newly predicted miRNAs, we analyzed their secondary structures using RNAfold. Stable hairpin formation and low minimum free energy are hallmark features of genuine miRNA precursors, essential for Dicer processing and subsequent mature miRNA function (Table 2). The structural profiles and positional entropy depicted in Fig. 3 demonstrate that the novel miRNAs, including novel_538 (which targets CNN2), exhibit canonical stem-loop configurations with favorable thermodynamic stability. This structural validation supports their identity as bona fide miRNAs and provides a foundation for interpreting their potential regulatory roles in the host–parasite interaction.

**Table 2:** The mature sequences, the position of the predicted miRNA precursor in the reference sequences (hairpin positions), and the free energy of the thermodynamic ensemble (free energy) values of novel miRNAs

	Mature fasta	Hairpin position	Free energy (kcal/mol)
novel_505sequence	cuuaugaguguagauacuguua	C1:78902532..78902584:−	−21.30
	CUAGUAUCAACACUCAUAAACUCUACACAGCUUAUGAGUGUAGAUACUGUUA 5′.(((((((.((((((((((.((……)).)))))))))).)))))))…3′		
novel_416sequence	uggggcuggagaaggucc	B4:133135438..133135478:+	−9.60
	AACUGAAGCCAUCAGCGGAGCUUGGGGCUGGAGAAGGUCC 5′..((..((((…(((…)))…))))..))……. 3′		
novel_279sequence	aaagugcucccauuuuugugugu	E2:4329278..4329336:−	−29.30
	ACACAAAAUGGGGGCACUUCCCGUUCUCUGUCUGGAAAGUGCUCCCAUUUUUGUGUGU 5′(((((((((((((((((((.(((………))).))))))))))).))))))))..3′		
novel_442sequence	uaugaauggugccuugcugagc	X:122985966..122986018:−	−26.90
	UCGGGAAGGCAUCAUUCACAUAGAUGGAUAUAUGAAUGGUGCCUUGCUGAGC 5′((((.(((((((((((((.(((……))).))))))))))))).))))..3′		
novel_370 sequence	uguugacugugucccgugcau	C1:13012336..13012411:+	−26.70
	UCCGGGGUCAAGGCCGGCAUGAGUCUGCCCAGCCUGGGAACACUAACAAACUCGUGUUGACUGUGUCCCGUGCAU 5′..(((((.((.((.((((((((((.(((((…..)))……..)).)))))))))).)).)))))))….. 3′		
novel_474 sequence	gccccaagugggacucugugcu	A1:30938437..30938477:+	−31.60
	GCCCCAAGUGGGACUCUGUGCUGAUGGCACGGAACCUGCUUGGGGUUC 5′((((((((..((..(((((((…..))))))).))..))))))))..3′		
novel_486 sequence	gaguucugggcuguagugg	A2:49518407..49518465:−	−18.60
	GCUACAGGAGGAACACUGCAUGUCUGAGGCUUAGGAAGAGAGUUCUGGGCUGUAGUGG 5′………….(((((((.(((((..((((…….))))..)))))))))))). 3′		
novel_469 sequence	uucacaguggcuaagugca	X:76795397..76795458:−	−26.60
	UUCACAGUGGCUAAGUGCAGUGUACUGCAGCAGCAGUGCACUCAAUGGUUGAUUGUGGCAU 5′.((((((((((((..((.((((((((((….)))))))))))).))))).)))))))… 3′		
novel_538 sequence	ucuuagcugagcgucgcu	C2:134814808..134814894:+	−22.84
	UCUUAGCUGAGCGUCGCUUCCAAGGGCAUCAACUUCAACCAAGCUGACUAAAAAGCCCAAUGGACUGCACCCCUUAGCAUUAGAGA 5′(((..((((((.(..((.((((.((((…….(((…….)))…….))))..))))..))..).))))))…)))..3′

### Interaction between miRNA and target mRNA

**Fig. 4:**
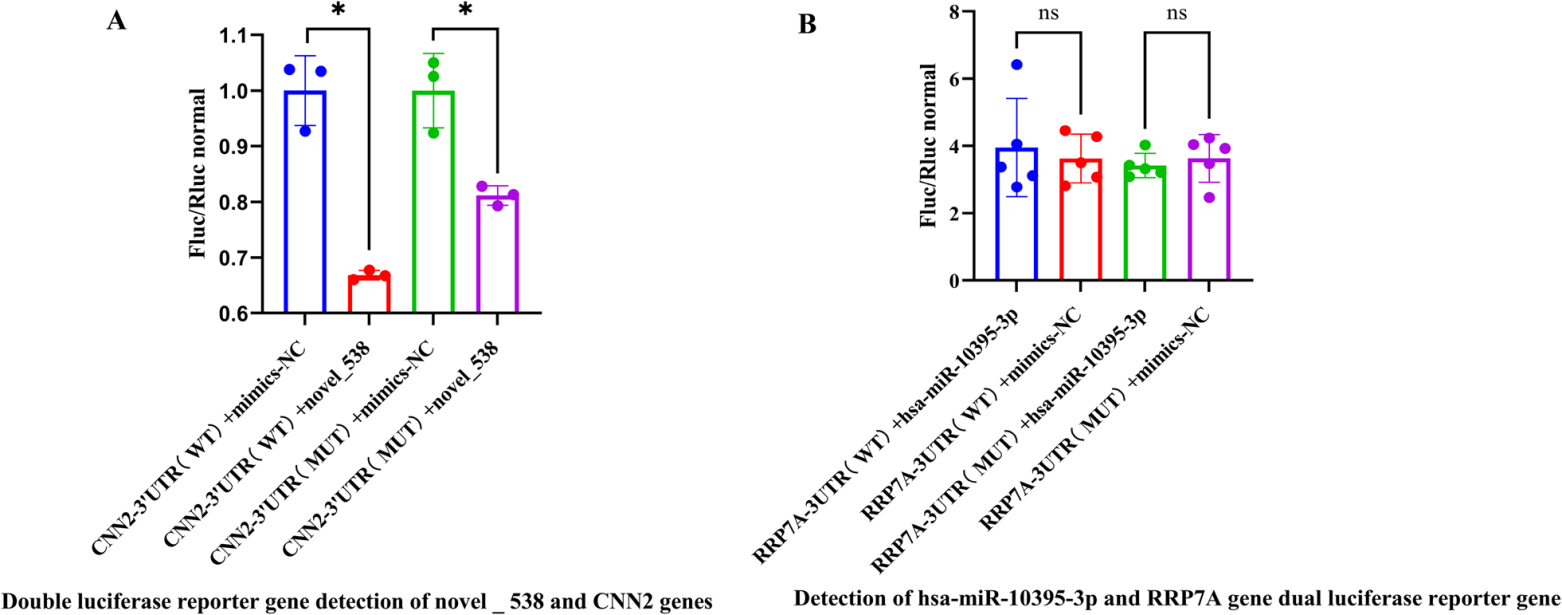
Dual-luciferase reporter assay of microRNA–target interactions **A** Novel_538 targeting *CNN2* 3′-UTR. **B** Hsa-miR-10395-3p targeting *RRP7A* 3′-UTR. The x-axis indicates the reporter construct type: wild-type (WT) or mutant (MUT) 3′-UTR, with mimics-NC as the negative control. The y-axis shows the normalized firefly/renilla luciferase activity (Fluc/Rluc). Data are presented as scatter plots with individual data points from three or more independent replicates (*n* ≥ 3). The wild type of the gene is represented by WT, and the mutant type is represented by MUT. Mimics-NC represents the control. * *P* < 0.05; ns indicates that the difference is not significant

To validate the predicted miRNA–target interactions and assess their potential functional relevance in *T. gondii* infection, we selected two miRNA–mRNA pairs for experimental verification: novel_538–*CNN2* and hsa-miR-10395-3p–*RRP7A*. These pairs were chosen based on their significant enrichment in pathways related to cytoskeletal organization and cellular metabolism, which are pivotal during parasite invasion and intracellular development. The identification of miRNA– target gene interactions was performed using dual-luciferase reporter assays (Additional File 6). As shown in the results (Fig. 4A), after co-transfection of novel_538 with the CNN2-3′UTR (WT/MUT) reporter plasmids into 293 T cells, the relative luciferase activity of the CNN2-3′UTR wild-type (WT) reporter showed a significant difference compared with the mimics-NC group (*P* < 0.05), indicating that novel_538 could regulate CNN2-3′UTR (WT) in this experiment. Moreover, a significant difference was also observed for the CNN2-3′UTR mutant-type (MUT) reporter compared with the mimics-NC group (*P* < 0.05), suggesting that *CNN2* may possess additional regulatory binding sites. In contrast, after co-transfection of hsa-miR-10395-3p with the RRP7A-3′UTR–(WT/MUT) reporter plasmids into 293 T cells, the relative luciferase activity of both RRP7A-3′UTR (WT) and RRP7A-3′UTR (MUT) reporters showed no significant difference compared with the mimics-NC group (Fig. 4B). This indicates that the predicted targeting relationship between hsa-miR-10395-3p and the *RRP7A* gene could not be verified in the present experiment, implying that there may be no direct interaction between them, or that further optimization of experimental conditions and validation of other potential binding sites are required for confirmation.

### Functional enrichment of target genes in biological processes and pathways

**Fig. 5:**
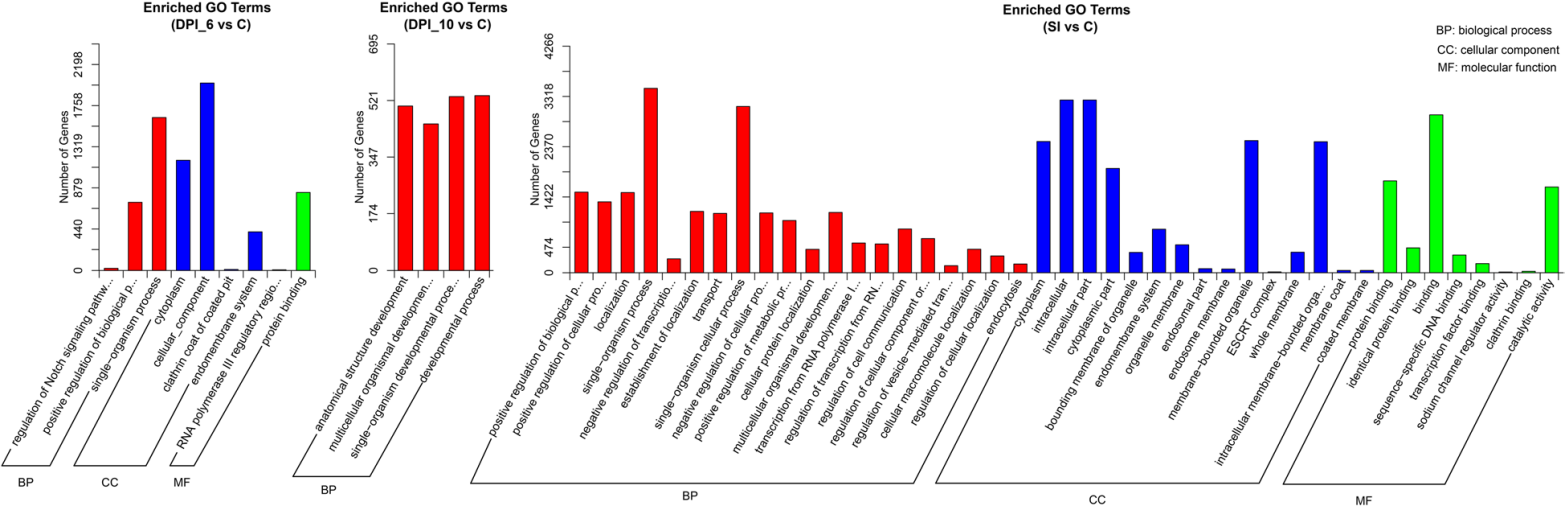
Gene Otology enrichment analyses on the sets of target genes for each group of DE miRNAs The bar graphs show the number of target genes enriched in GO terms belonging to the three GO categories, biological process (red), cellular component (blue) and molecular function (green). The x-axis represents the three GO terms and the y-axis represents the number of target genes

Due to the limited number of candidate target genes matched by DE miRNAs, certain GO categories (molecular function [MF]/cellular component [CC]) were absent at some infection time points (10 DPI). Moreover, no significantly enriched GO terms were identified at 14 DPI. This is likely attributable to the reduced number of DE miRNAs at this time point (only 36 DE miRNAs, Table S3), which corresponds to the later stage of primary infection when oocyst shedding has largely ceased and the host transcriptional response may have stabilized. In the biological process (BP) category, single-organism process, single-organism developmental process, developmental process, and single-organism cellular process were significantly enriched. For CC, cellular component, intracellular, and intracellular part were notably enriched. Under MF, binding and protein binding showed significant enrichment (Fig. 5).

**Fig. 6:**
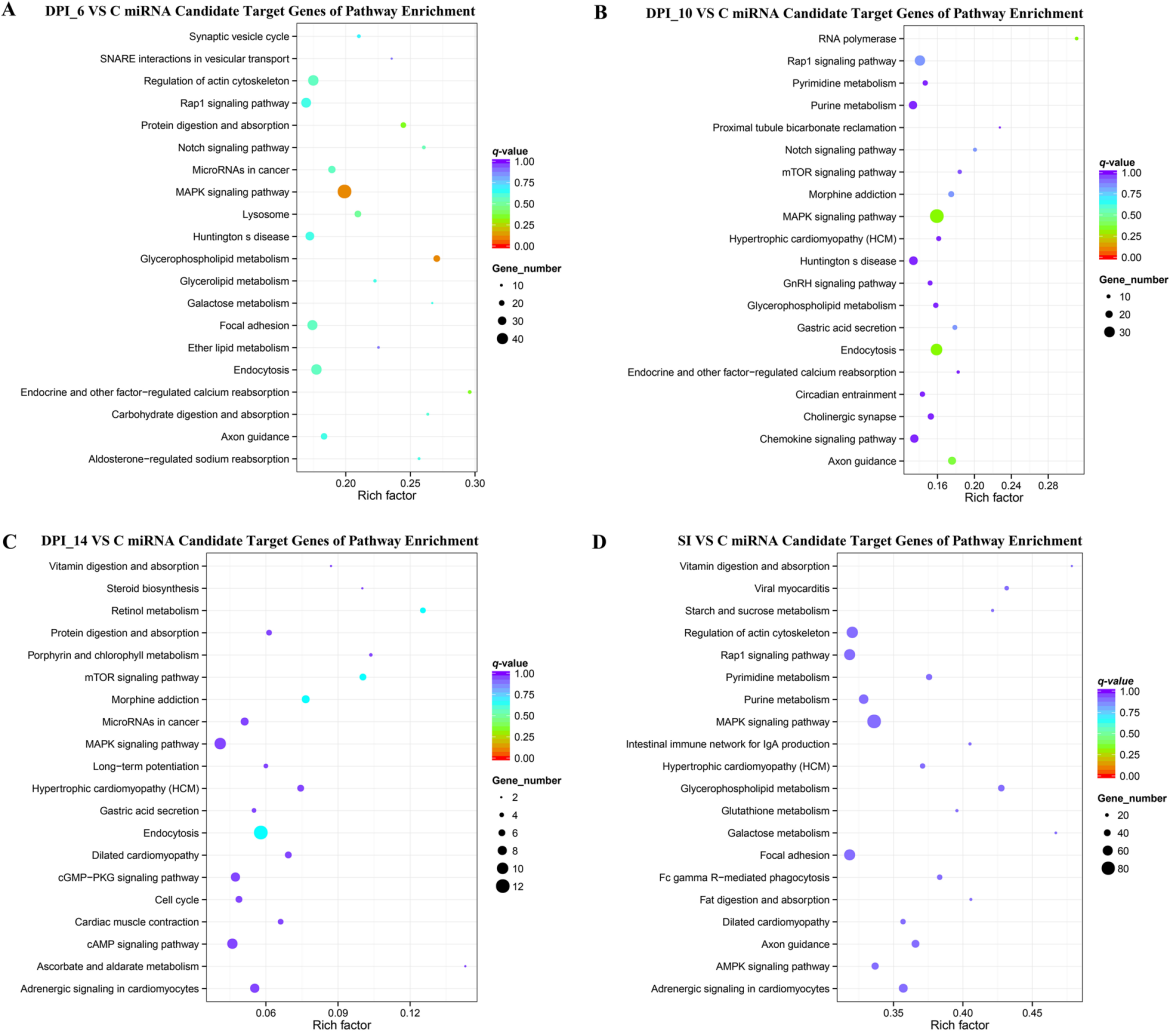
DE miRNA candidate target genes of pathway enrichment. The 20 most enriched KEGG pathways at 6, 10, and 14 days post-infection (DPI), and secondary infection (SI) at 27 days post-infection (DPI). **A**–**D** represents 6,10,14 and SI DPI enriched KEGG pathway, respectively. The y-axis represents the distinct KEGG pathways, while the x-axis represents the rich factor. The rich factor refers to the ratio of the target gene annotated in the pathway to the total number of genes annotated in the pathway. The higher the rich factor, the greater the degree of pathway enrichment. The size of the dots corresponds to the number of target genes, with larger dots denoting a larger number of target genes and vice versa. The colors of the dots represent the *P*-values (*P* < 0.05) of enrichment

Pathway enrichment analysis can identify the primary biochemical metabolic pathways and signal transduction pathways in which candidate target genes are involved. Among the four infection time points, the Rap1 and AMPK signaling pathways were significantly enriched, followed by miRNAs in cancer and regulation of actin cytoskeleton (Fig. 6).

### qPCR validation confirms sequencing results

**Fig. 7:**
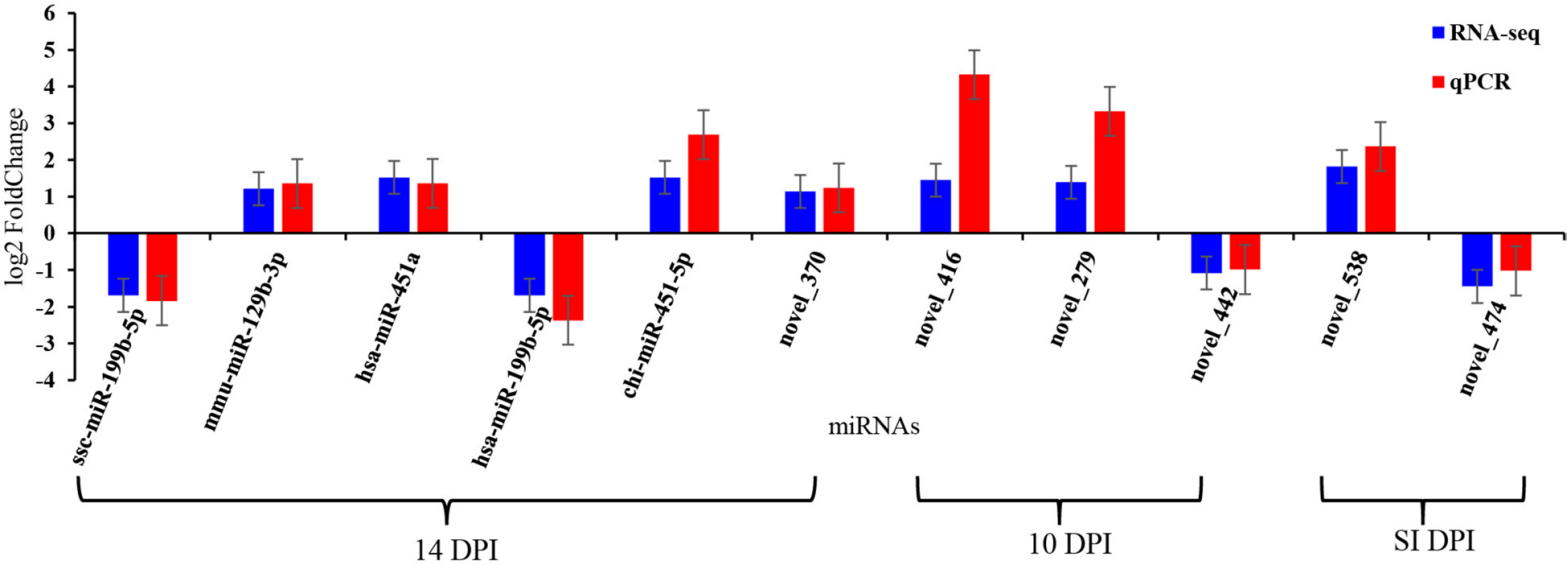
Verification of RNA-Seq results by qPCR verification of the RNA-Seq data using qPCR. Bars represent the mean fold changes in the expression of 11 *T. gondii* miRNAs

Eleven miRNAs (hsa-mir-451a, hsa-miR-199b-5p, ssc-mir-199b, mmu-mir-129b, chi-miR-451-5p, novel_474, novel_538, novel_279, novel_442, novel_370, and novel_416) were chosen for qPCR analysis with miRNA-specific primers to verify the expression levels of the DE miRNAs (Fig. 7). These findings confirm that the RNA-Seq data are stable and reliable.

## Discussion

Studies have shown that *T. gondii* infection can alter the expression patterns of miRNAs in hosts such as pigs and mice, suggesting that miRNAs are involved in the pathogenesis of toxoplasmosis [6, 11, 15–18]. Although miRNA expression in the liver of cats—the definitive host of *T. gondii*—has been reported [9], the role of miRNAs in the feline small intestine, the exclusive site of oocyst development, remains unexplored. Given that miRNAs are likely to regulate the development and shedding of oocysts, it is essential to investigate miRNA expression profiles in the small intestine of cats following *T. gondii* infection to elucidate their impact on parasite development. This study represents the first comprehensive investigation into the expression profiles and potential regulatory roles of miRNAs in the feline small intestinal epithelium during *T. gondii* infection. Our findings reveal dynamic alterations in the small RNA landscape, with a significant number of DE miRNAs identified across primary and secondary infection time points. These changes suggest an active role for miRNAs in the host–parasite interaction within the definitive feline host. The successful establishment of the *T. gondii* infection model, confirmed molecularly by *B1* gene PCR, allowed for reliable profiling of the host’s transcriptional response across primary (6, 10, 14 DPI) and secondary (SI DPI) infection stages.

The sequencing effort identified a remarkably rich miRNA repertoire of 2666 miRNAs, including 91 novel candidates, substantially expanding the known miRNA landscape in feline intestinal tissue. This number exceeds those reported in other *T. gondii*-infected tissues such as porcine spleen (252 miRNAs) or feline liver (384 miRNAs), highlighting the unique and active regulatory environment of the definitive host’s intestine [9, 11, 16, 18]. The high degree of similarity in expression profiles among biological replicates underscores the robustness of our experimental approach.

The temporal pattern of DE miRNAs is particularly revealing. The significantly higher number of DE miRNAs at 6 DPI (126) and 10 DPI (122), compared to the notable decline at 14 DPI (36) (Fig. 1), temporally coincides with the established biological timeline of active oocyst production and shedding in felids, with shedding predominantly occurring between 3 and 10 DPI [19, 20]. Therefore, the peak of miRNA dysregulation coincides with the period of active parasite sexual replication and initial oocyst release, suggesting these miRNAs are integral components of the host–parasite interface during this critical developmental window. The subsequent surge in DE miRNAs during secondary infection (237 at SI DPI) points toward a potent and distinct memory or amplified regulatory response upon re-challenge.

Several DE miRNAs identified in this study, including mmu-miR-142a-3p, mmu-miR-365-3p, mmu-let-7 g-5p, mmu-miR-146a-5p, mmu-miR-125a-3p, and mmu-miR-150-5p, have also been reported in previous research by other investigators [9, 11, 21]. This overlap indicates that the feline miRNAs identified here are similarly dysregulated in intermediate hosts of *T. gondii*. Notably, hsa-miR-199b-5p, ssc-miR-199b-5p, mmu-miR-129b-3p, and mmu-miR-129-5p were consistently dysregulated across multiple infection stages. The persistent downregulation of hsa-miR-199b-5p and ssc-miR-199b-5p at 6, 10, and 14 DPI suggests their potential involvement in critical early host responses or parasite-driven modulation mechanisms. Their mutual regulation via *CYTH1* and *COQ7* mRNAs points to a coordinated regulatory network that may influence host cell processes beneficial to parasite survival or development. Based on existing research, miR-199b-5p (downregulated) has been shown to inhibit apical-basolateral polarity in oral squamous cell carcinoma by targeting Scribble/Lgl [22]. Meanwhile, by targeting AKT1, miR-199b-5p may influence the PI3K/AKT pathway—a key pathological pathway in sarcoidosis. Furthermore, miR-199b-5p (downregulated) has been identified as a critical miRNA significantly associated with sarcoidosis, suggesting its potential as a biomarker for distinguishing patients from healthy controls [23]. In triple-negative breast cancer, miR-199b-5p was found to significantly suppress cell proliferation and invasion, functioning as a potential tumor suppressor. Its downregulation may lead to the loss of inhibition on target genes such as N-cadherin, potentially promoting abnormal cellular processes [24]. Building on these findings, we propose that the persistent downregulation of miR-199b-5p may facilitate *T. gondii* sexual reproduction by coordinately relieving suppression of its target genes *CYTH1* and *COQ7*. This could promote a permissive intestinal niche through multiple mechanisms: *CYTH1* upregulation may alter epithelial cytoskeleton and cell adhesion, potentially aiding parasite invasion and intracellular niche formation; *COQ7* induction could rewire host mitochondrial metabolism to support parasite energy demands. Together, these changes likely converge on key pathways such as Rap1 and AMPK signaling, collectively reshaping host cell architecture, metabolism, and signaling to favor gametogenesis and oocyst production. Thus, this study unveils a previously unrecognized layer of post-transcriptional regulation through which *T. gondii* orchestrates host cell processes to favor oocyst development in its definitive feline host.

Cluster analyses reinforced the existence of co-regulated miRNA modules responding to infection. The prominence of DE miRNAs homologous to those from *M. musculus* and *H. sapiens* not only supports evolutionary conservation but also implies that findings in this feline model may have translational relevance for understanding miRNA roles in human and other animal infections [15].

The predicted miRNA–mRNA interaction networks revealed both broad and specific regulators. miRNAs like rno-miR-361-3p (targeting 53 mRNAs at 6 DPI) and hsa-miR-320a-3p (targeting 24 mRNAs at SI DPI) appear to function as master regulators, potentially orchestrating large-scale changes in host cell processes. Notably, hsa-miR-320a is implicated in various diseases, including its role as a tumor suppressor. Its significant downregulation and broad target set here suggest it may be a crucial regulator of pathways hijacked by *T. gondii* [25, 26]. The involvement of novel miRNAs, such as novel_538 targeting *CNN2* (calponin 2, an actin-binding protein) and novel_474 targeting *ABCC5* (an efflux transporter), points to the discovery of previously uncharacterized regulatory axes in the host–parasite interaction. The dual-luciferase assay partially validated these in silico predictions; while the novel_538–*CNN2* interaction was supported, the lack of confirmation for hsa-miR-10395-3p–*RRP7A* underscores the need for combinatorial validation approaches and suggests potential indirect or condition-specific regulation.

Functional enrichment analysis of the predicted target genes provided crucial insights into the biological processes and pathways likely influenced by the DE miRNAs. The significant enrichment in GO terms related to “developmental process,” “single-organism cellular process,” and “binding” underscores a targeted manipulation of fundamental host cell functions. The KEGG pathway analysis was particularly informative. The consistent enrichment of the Rap1 and AMPK signaling pathways across time points is highly significant. The Rap1 pathway is central to cell adhesion, junction formation, and polarity processes critical for maintaining intestinal epithelial barrier integrity [27]. Its dysregulation could facilitate parasite invasion or disrupt tissue architecture. The AMPK pathway is a key sensor and regulator of cellular energy homeostasis [28]. The Rap1 and AMPK signaling pathways are highly conserved across eukaryotic cells, where they participate in the regulation of cell adhesion, polarity, and metabolism. These pathways are likely to represent common regulatory targets exploited by *T. gondii* across different host species. Thus, the discovery of these conserved molecules and pathways provides important clues for understanding shared regulatory strategies employed by *T. gondii* in diverse hosts. Furthermore, the enrichment of pathways like “MicroRNAs in cancer” and “Regulation of actin cytoskeleton” aligns with the well-established strategies of intracellular pathogens like *T. gondii*, which often manipulate host cell proliferation, motility, and structural dynamics for survival and immune evasion. While our study provides the first miRNA landscape in the definitive host’s intestine and identifies these pathways as major targets of dysregulated miRNAs, we acknowledge that these are predictive associations. Future functional studies, focusing on validating the specific miRNA–mRNA interactions within these pathways (such as the novel_538–*CNN2* axis related to the actin cytoskeleton), are essential to confirm their precise role in the host–parasite interplay during oocyst development.

## Conclusions

This study reveals a dynamic and complex landscape of miRNA expression in the feline small intestine during *T. gondii* infection. The temporal correlation of miRNA dysregulation with oocyst shedding, the identification of consistently altered key miRNAs, and the enrichment of specific pathways collectively suggests that *T. gondii* orchestrates a sophisticated post-transcriptional regulatory program in its definitive host. This program likely aims to modify the intestinal epithelial environment to favor parasite sexual reproduction and oocyst production. Future functional studies focusing on the validated targets and the implicated signaling pathways are essential to dissect the precise mechanisms by which these miRNAs influence the parasite’s life cycle and to evaluate their potential as targets for intervention strategies aimed at disrupting oocyst shedding and environmental contamination.

## Supplementary Information

**Additional file 1**. Table S1. The clean reads data of miRNA

**Additional file 2**. Table S2. Enrichment information of miRNA

**Additional file 3**. Table S3. Differentially expressed miRNA information

**Additional file 4**. Figure S1. Expression and correlation between miRNA groups. **A** TPM density distribution diagram. The abscissa is the log10value of miRNA, and the ordinate is the density corresponding to log10. **B** TPM box plots of different groups. The abscissa is the name of the group, the ordinate is log10, and the box plot of each region is five statistics. **C** The correlation diagram of miRNA expression between samples. The abscissa and ordinate are log10

**Additional file 5**. Figure S2. Differential miRNA clustering and species analysis. **A** Differential miRNA clustering diagram, overall hierarchical clustering diagram, clustering with log10value, red indicates high-expression miRNA, blue indicates low-expression miRNA. **B** K_means_cluster clustering diagram, which is clustered with the relative expression level of miRNA log_2_. The gray lines in each subgraph represent a line chart of the relative expression of miRNAs in a cluster under different experimental conditions, and the blue lines represent a line chart of the average relative expression of all miRNAs in this cluster under different experimental conditions. The red line is the reference, the on-line is upregulated, and the off-line is downregulated. The x-axis represents the experimental conditions, and the y-axis represents the relative expression level. **C** SOM_cluster clustering diagram, which is clustered by the relative expression level of miRNA log_2_. The gray lines in each subgraph represent the relative expression of miRNAs in a cluster under different experimental conditions, and the blue lines represent the average relative expression of all miRNAs in this cluster under different experimental conditions. The red line is used as a reference, the line is upregulated, and the line is downregulated. The x-axis represents the experimental conditions, and the y-axis represents the relative expression. **D** Analysis of differentially expressed miRNA families. The x-axis is the number of miRNAs in each family, and the y-axis is the type of miRNA family

**Additional file 6**. Dual-luciferase reporter assays

## Data Availability

The datasets supporting the findings of this article are included within the paper. The RNA-Seq data obtained in this study have been deposited in the Mendeley Data database, and our data also uploaded (10.17632/jmjyx6xpx5.1).

## Abbreviations

BP: Biological process
CC: Cellular component
MF: Molecular function
DE: Differentially expressed
DPI: Days post-infection
GO: Gene Ontology
KEGG: Kyoto Encyclopedia of Genes and Genomes
miRNA: MicroRNA
PCR: Polymerase chain reaction
RNA-Seq: RNA sequencing
siRNA: Small interfering RNA
snoRNA: Small nucleolar RNA
TPM: Transcripts per million
UTR: Untranslated region
ELISA: Enzyme-linked immunosorbent assay
DESeq: Differential expression analysis for sequence data
