# Supplementary figures and images for: Dynamic landscape of microRNA expression in the feline small intestine during Toxoplasma gondii infection
Source: Parasit Vectors. 2026 Apr 11;19:220. doi: 10.1186/s13071-026-07356-7 (PMC13185173; doi:10.1186/s13071-026-07356-7)

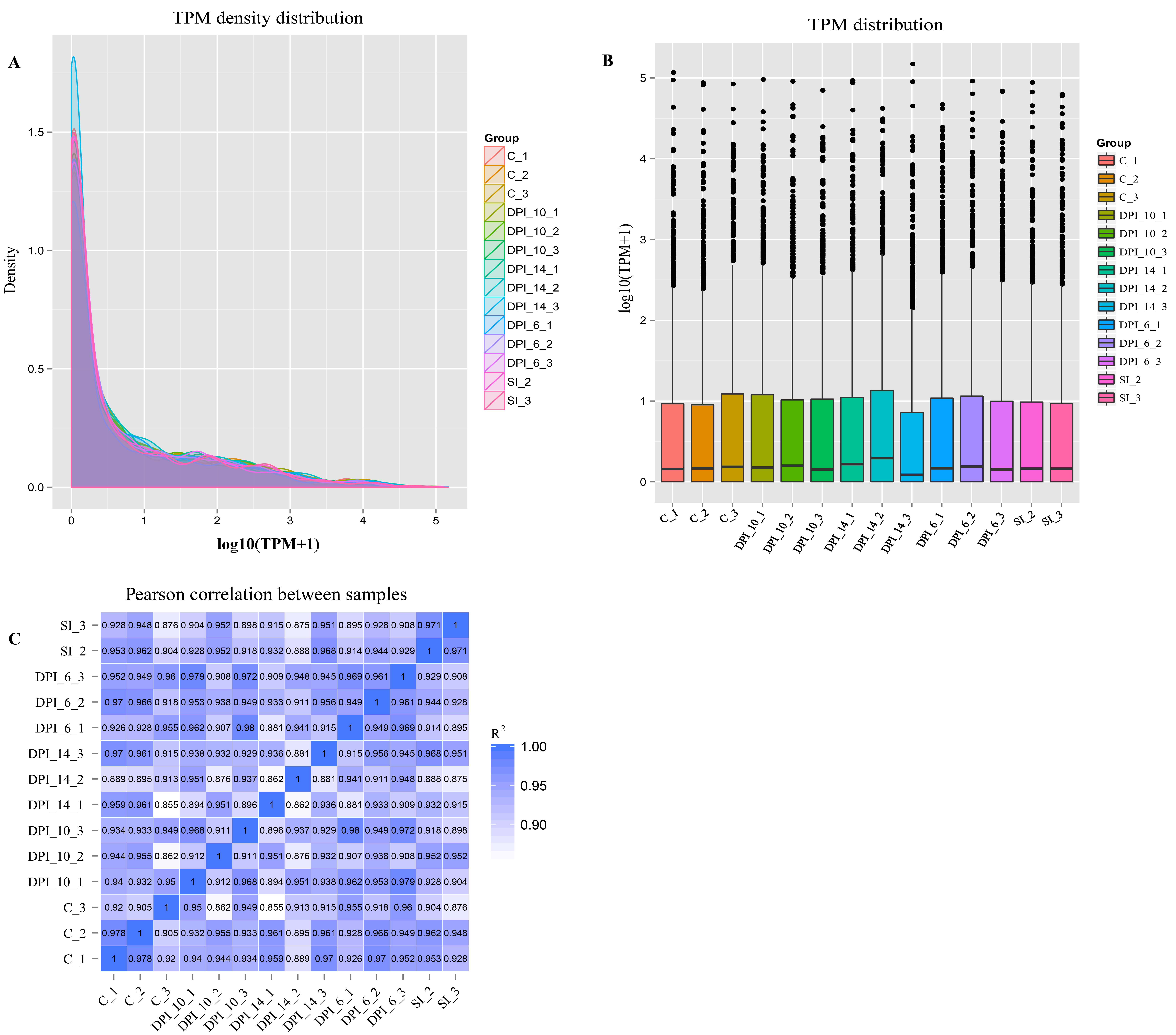

Supplement: Supplementary file 4 — Additional file 4. Figure S1. Expression and correlation between miRNA groups. A TPM density distribution diagram. The abscissa is the log10value of miRNA, and the ordinate is the density corresponding to log10. B TPM box plots of different groups. The abscissa is the name of the group, the ordinate is log10, and the box plot of each region is five statistics. C The correlation diagram of miRNA expression between samples. The abscissa and ordinate are log10 [file 13071_2026_7356_MOESM4_ESM.tif]
