# Supplementary material for: Dynamic landscape of microRNA expression in the feline small intestine during Toxoplasma gondii infection
Source: Parasit Vectors. 2026 Apr 11;19:220. doi: 10.1186/s13071-026-07356-7 (PMC13185173; doi:10.1186/s13071-026-07356-7)
Supplement: Supplementary file 5 — Additional file 5. Figure S2. Differential miRNA clustering and species analysis. A Differential miRNA clustering diagram, overall hierarchical clustering diagram, clustering with log10value, red indicates high-expression miRNA, blue indicates low-expression miRNA. B K_means_cluster clustering diagram, which is clustered with the relative expression level of miRNA log2. The gray lines in each subgraph represent a line chart of the relative expression of miRNAs in a cluster under different experimental conditions, and the blue lines represent a line chart of the average relative expression of all miRNAs in this cluster under different experimental conditions. The red line is the reference, the on-line is upregulated, and the off-line is downregulated. The x-axis represents the experimental conditions, and the y-axis represents the relative expression level. C SOM_cluster clustering diagram, which is clustered by the relative expression level of miRNA log2. The gray lines in each subgraph represent the relative expression of miRNAs in a cluster under different experimental conditions, and the blue lines represent the average relative expression of all miRNAs in this cluster under different experimental conditions. The red line is used as a reference, the line is upregulated, and the line is downregulated. The x-axis represents the experimental conditions, and the y-axis represents the relative expression. D Analysis of differentially expressed miRNA families. The x-axis is the number of miRNAs in each family, and the y-axis is the type of miRNA family [file 13071_2026_7356_MOESM5_ESM.doc]

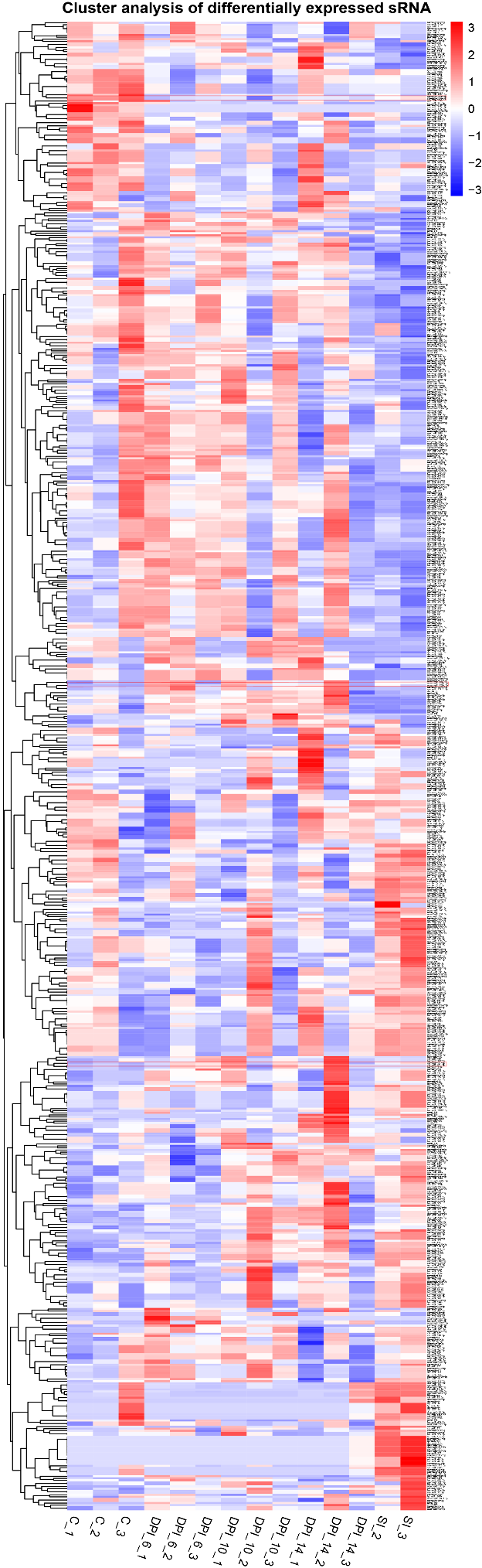


Additional File 5 Figure S2A


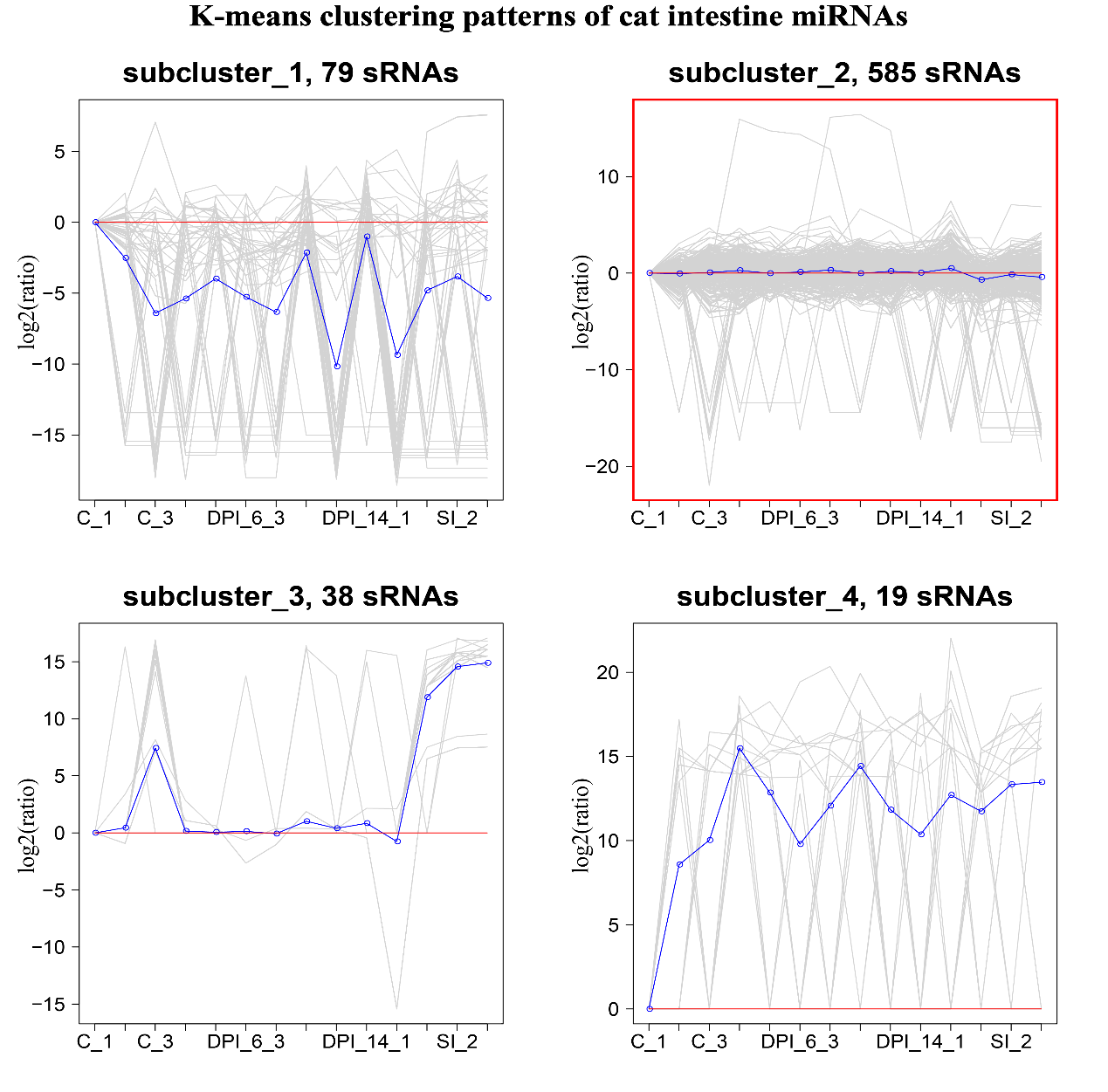


Additional File 5 Figure S2B
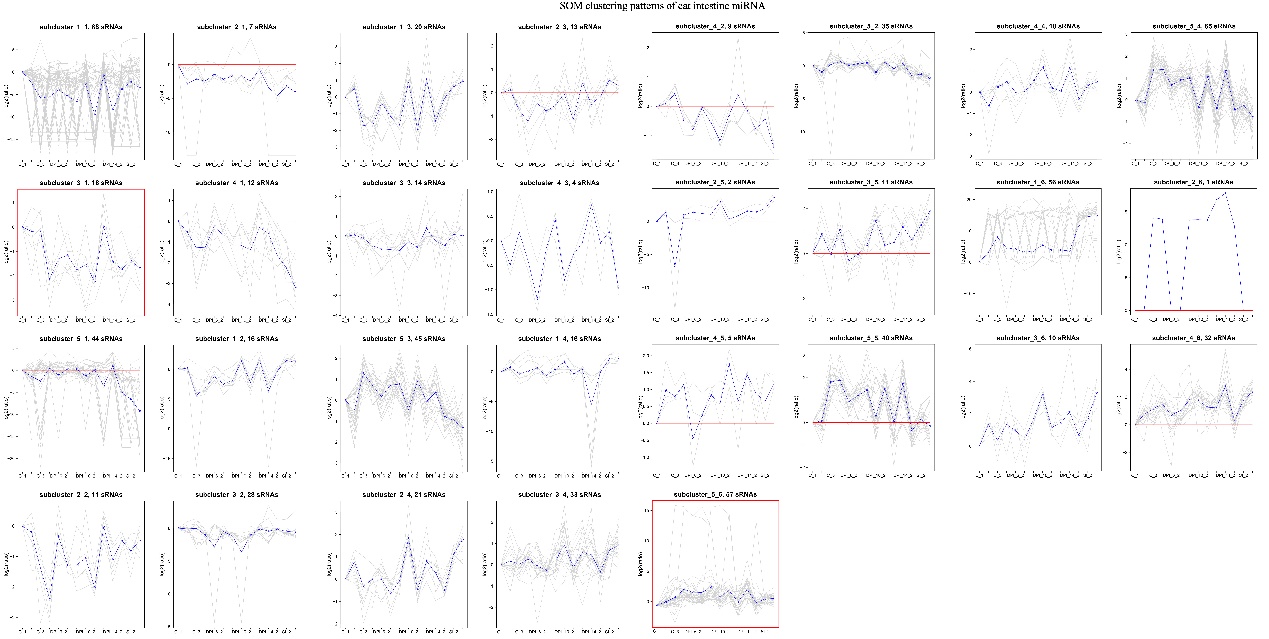
 Additional File 5 Figure S2C
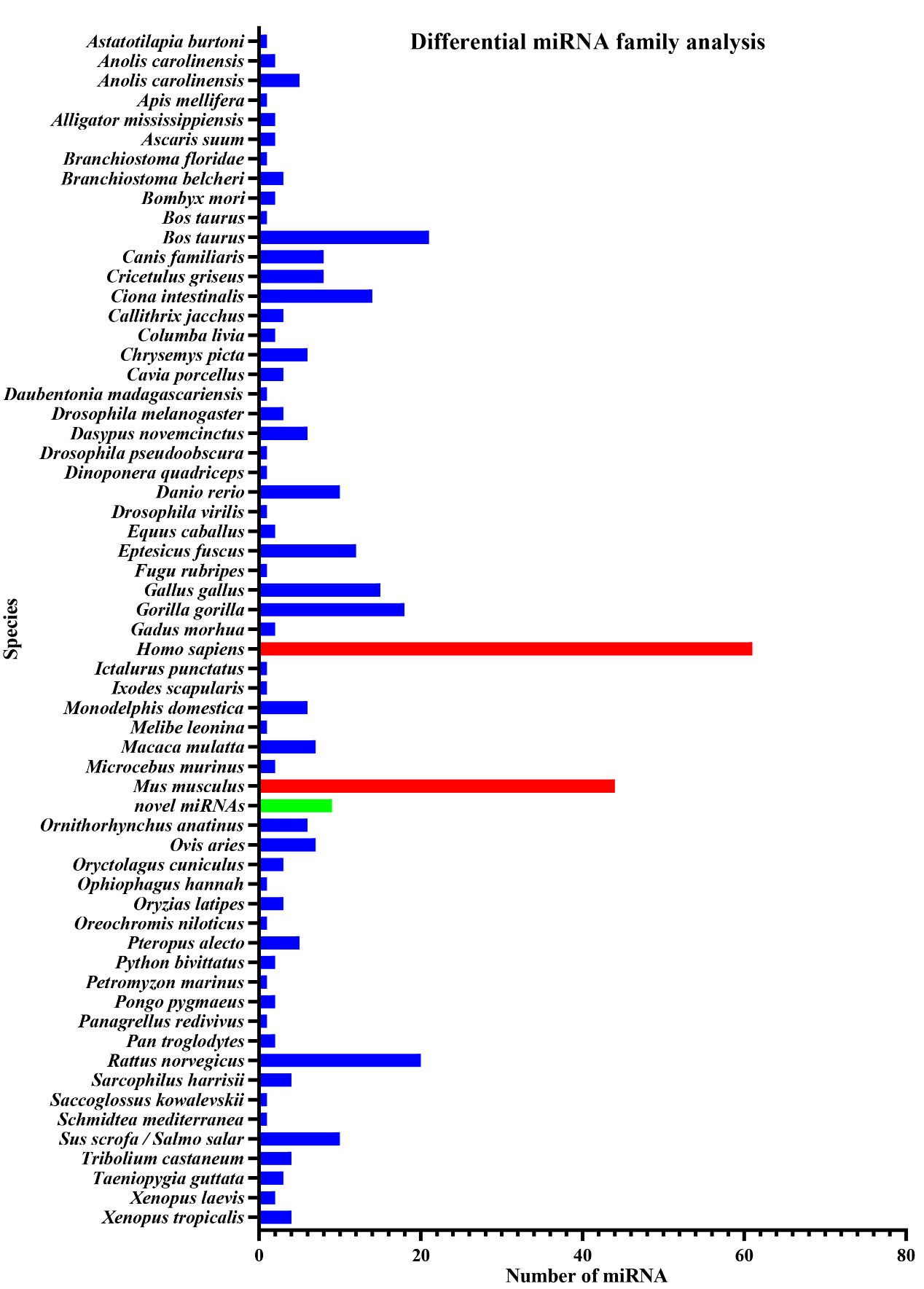


Additional File 5 Figure S2D
