# Supplementary material for: Dynamic landscape of microRNA expression in the feline small intestine during Toxoplasma gondii infection
Source: Parasit Vectors. 2026 Apr 11;19:220. doi: 10.1186/s13071-026-07356-7 (PMC13185173; doi:10.1186/s13071-026-07356-7)
Supplement: Supplementary file 6 — Additional file 6. Dual-luciferase reporter assays [file 13071_2026_7356_MOESM6_ESM.docx]

**Dual-Lluciferase Reporter Assay for Investigating microRNA-Target mRNA Interactions**

**I. Vector Construction**

Information on the Vector and Target Gene

In this experiment, the pmirGLO vector was used. The vector map is shown below, with the target fragment inserted at the restriction enzyme site:

| 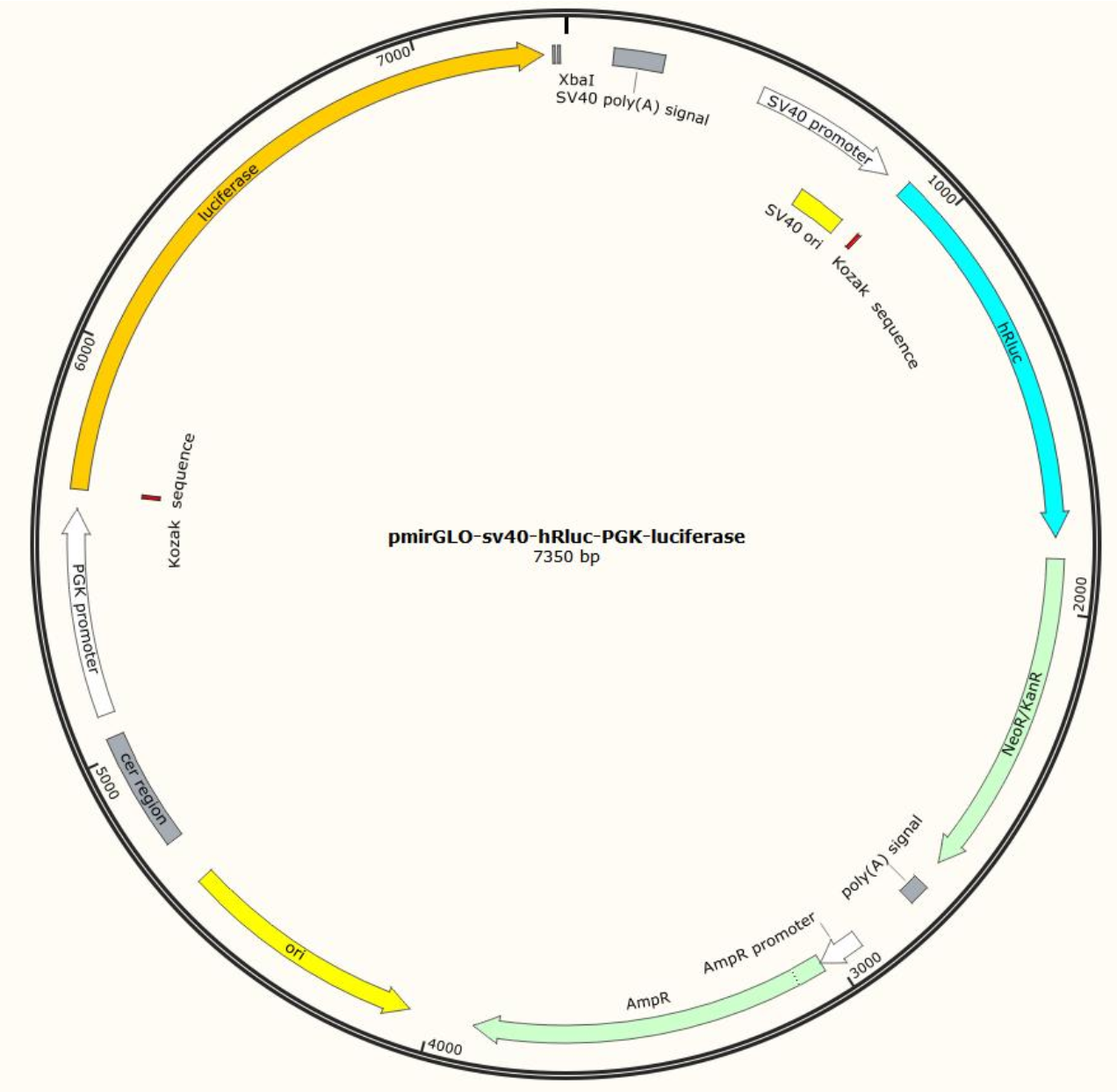 |
| --- |
|  |

The DNA sequence information of the target gene is as follows:

pmirGLO CNN2-3’UTR(WT)
ggctggcctggggggtggaggtgttgggggaagaaacctgggcagggaggggccctgccccgtatggtttccggtcgcctttccctctcttcccttttctctgccgatcagtttgtggtttctgtgcccacagaagatttgggcagttttaatgaaagaaaaataatatttttttgcaggggagtggggggcggggtttggagatggtggaaggggatgctgccgagaaggaagcaggtcccctgggagagggcagggccacagtccccaagtattttaggtcttgtgaggacctagcagtctcagacagtcacccgtagcccccccctccccccccccccagggcctagcggagaaactgaggcccaggcaggacaacagagttatgcctggttgacac

pmirGLO CNN2-3’UTR(MUT)

ggctggcctggggggtggaggtgttgggggaagaaacctgggcagggaggggccctgccccgtatggtttccggtcgcctttccctctcttcccttttctctgccgatcagtttgtggtttctgtgcccacagaagatttgggcagttttaatgaaagaaaaataatatttttttgcaggggagtggggggcggggtttggagatggtggaattgtcgtagtactctcaggaagcaggtcccctgggagagggcagggccacagtccccaagtattttaggtcttgtgaggacctagcagtctcagacagtcacccgtagcccccccctccccccccccccagggcctagcggagaaactgaggcccaggcaggacaacagagttatgcctggttgacac

pmirGLO RRP7A-3’UTR(WT):

gggtgaacgacagctcctcccggggccctggtgctcctgcagtctcgggaccccatgctggcggggcccgcccggggtgagggcttgtcaggacgccaccagcctggggagcgcttgctccctgctcatctctgttcccggcctgttcttggactctcctctcaggctaagtgtctgctgtgttctccgcttcccccattggaagggcccccgctggcagcgaaacggcctcggaaacagtgcgaatacagaacattttatttttcatccagctgggcagcaacctggcccttccttcctccgtccgctacccattcactgccgattatcagccacaaaacagacaaccctccaggtccaaacagtgtctcccccttgaagtctcggataccaaaagtcggaattttggccccatccctccaaaccctgccccaggtttctctgcctcagcccctgctggtctccaagacccgcgaggcctgtccccaccgcacctggcc

pmirGLO RRP7A-3’UTR(MUT):

gggtgaacgacagctcctcccggggccctggtgctcctgcagtctcgggaccccatgctggcggggcccgcccggggtgagggcttgtcaggacgccaccagcctggggagcgcttgctccctgctcatctctgttcccggcctgttcttggactctcctctcaggctaagtgtctgctgtgttctccgcttcccccattggaagggcccccgctggcagcgaaacggcctcggaagaacattttatttttcatccagctgggcagcaacctggcccttccttcctccgtccgctacccattcactgccgattatcagccacaaaacagacaaccctccaggtccaaacagtgtctcccccttgaagtctcggataccaaaagtcggaattttggccccatccctccaaaccctgccccaggtttctctgcctcagcccctgctggtctccaagacccgcgaggcctgtccccaccgcacctggcc

**2. Integration of target prediction and purpose fragment synthesis**

| 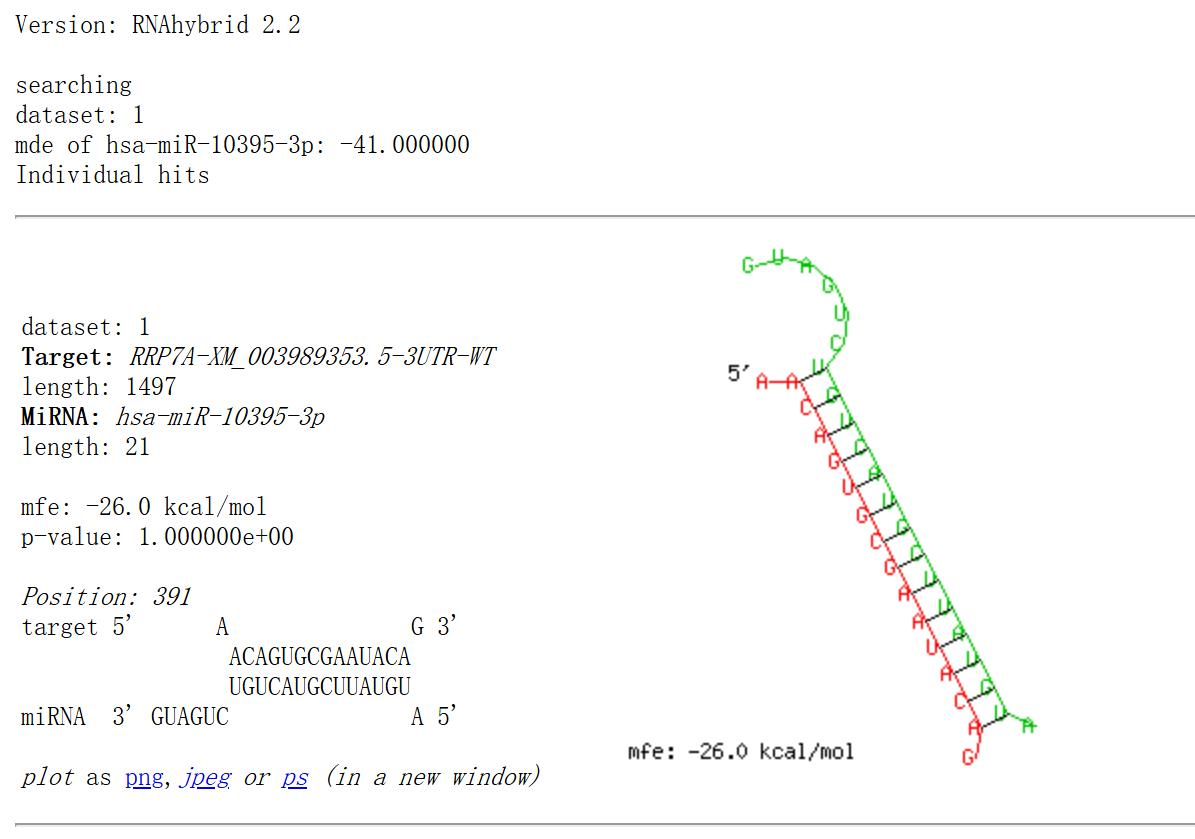 |
| --- |
| Prediction of binding sites of RRP7A-3’UTR(WT) and hsa-miR-10395-3p |

| 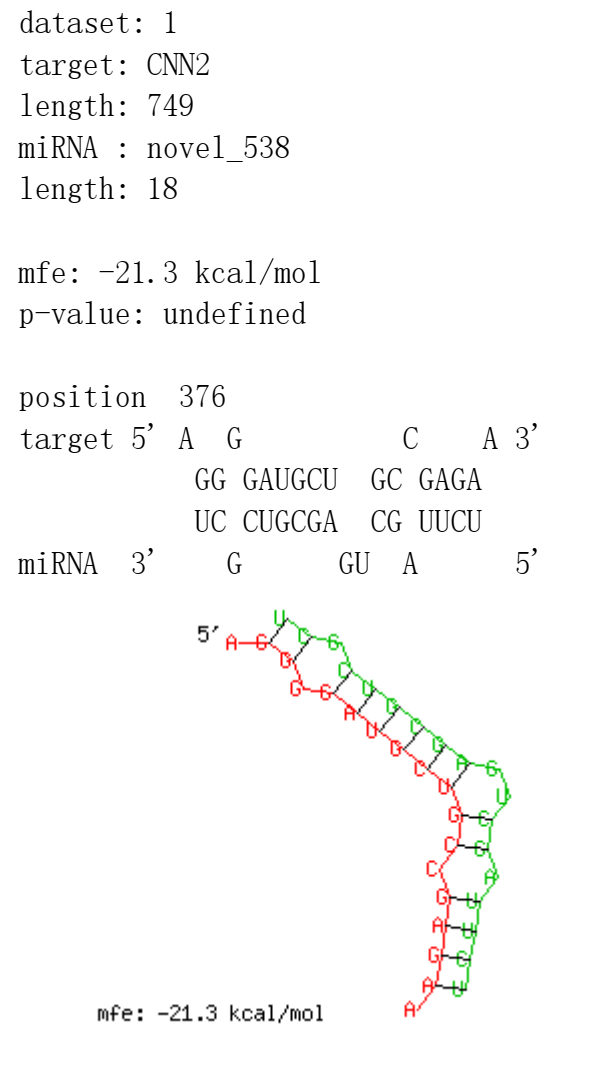 |
| --- |
| Prediction of binding sites of CNN2-3’UTR(WT) and novel_538 |

**3. Experimental Workflow for Molecular Cloning**

Construction of PmirGLO‑WT/MUT

The primers, synthesized by Sangon Biotech (Shanghai), were diluted to 10 µM each. The PmirGLO vector was digested with double restriction enzymes, followed by gel purification of the digested product. The target DNA fragments were also synthesized by Sangon Biotech. These fragments were amplified by PCR and then ligated into the digested vector. The ligation product was transformed into TOP10 competent cells (Ampicillin resistance) and incubated overnight at 37 °C.

The following day, single colonies were picked and inoculated into 5 mL of LB medium containing ampicillin, followed by shaking at 220 rpm for 16 hours at 37 °C. Plasmids were extracted using a miniprep kit and sent for commercial sequencing. Clones with correct sequencing results were selected for large‑scale plasmid preparation with endotoxin removal.

**4. Procedures for Cell Transfection**

1) One day prior to transfection, plate 293T cells in a 24-well plate (6×10⁴ cells per well) according to the required experimental groups and replicates. When cell confluency reaches 50%–70%, proceed with transfection.

2) Prepare Solution A by thoroughly mixing 50 μL of DMEM with 0.4 μg of WT/MUT target plasmid and 1 μL of 20 pmol/μL mimics/Negative Control (N.C.), then incubate at room temperature. Separately, prepare Solution B by mixing 48 μL of DMEM with 2 μL of lipo2000 transfection reagent without the need for room temperature incubation.

3) Combine Solution A and Solution B completely and allow the mixture to stand at room temperature for 30 minutes.

4) Add the transfection mixture into the 24-well plate and gently swirl to mix evenly. Return the plate to the incubator for continued culture.

5) Replace the medium with fresh culture medium 6 hours after transfection.

6) Perform luciferase assays 24-48 hours post-transfection.

**5. Experimental Procedure for Detection**

1) Remove the old culture medium and wash once with PBS.

2) Add 200 µL of 1X cell lysis buffer, shake for 15 minutes, and then transfer the lysate to a 1.5 mL tube by pipetting.

3) Centrifuge at 12,000 rpm for 10 minutes, and transfer 20 µL of the supernatant to a white enzyme-linked immunosorbent assay (ELISA) plate.

4) Add 100 µL of Firefly luciferase substrate working solution equilibrated to room temperature, mix thoroughly, and measure the luminescence.

5) Add 100 µL of Renilla luciferase substrate working solution and measure the luminescence.

**6. Result**

| 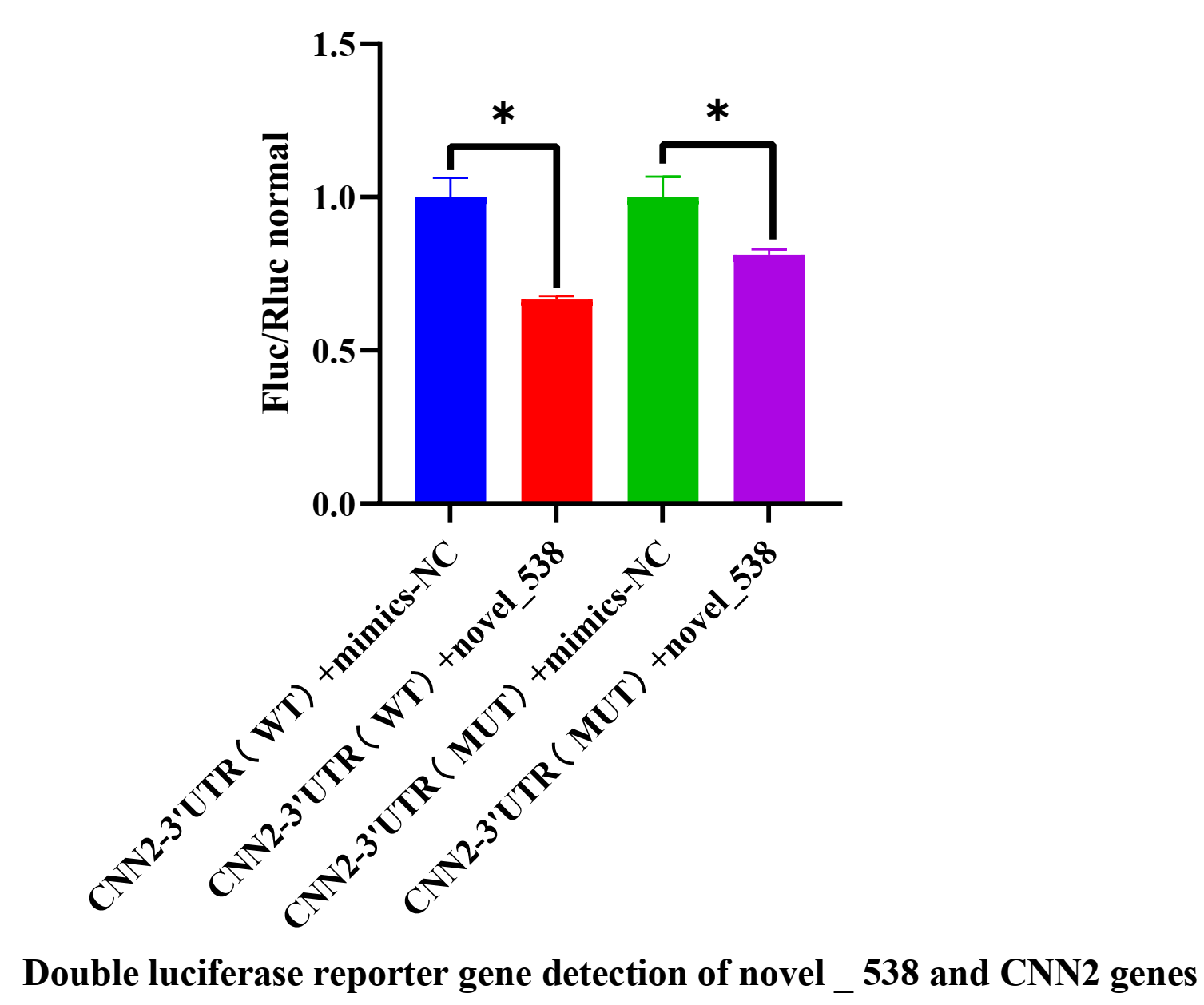 |
| --- |
| 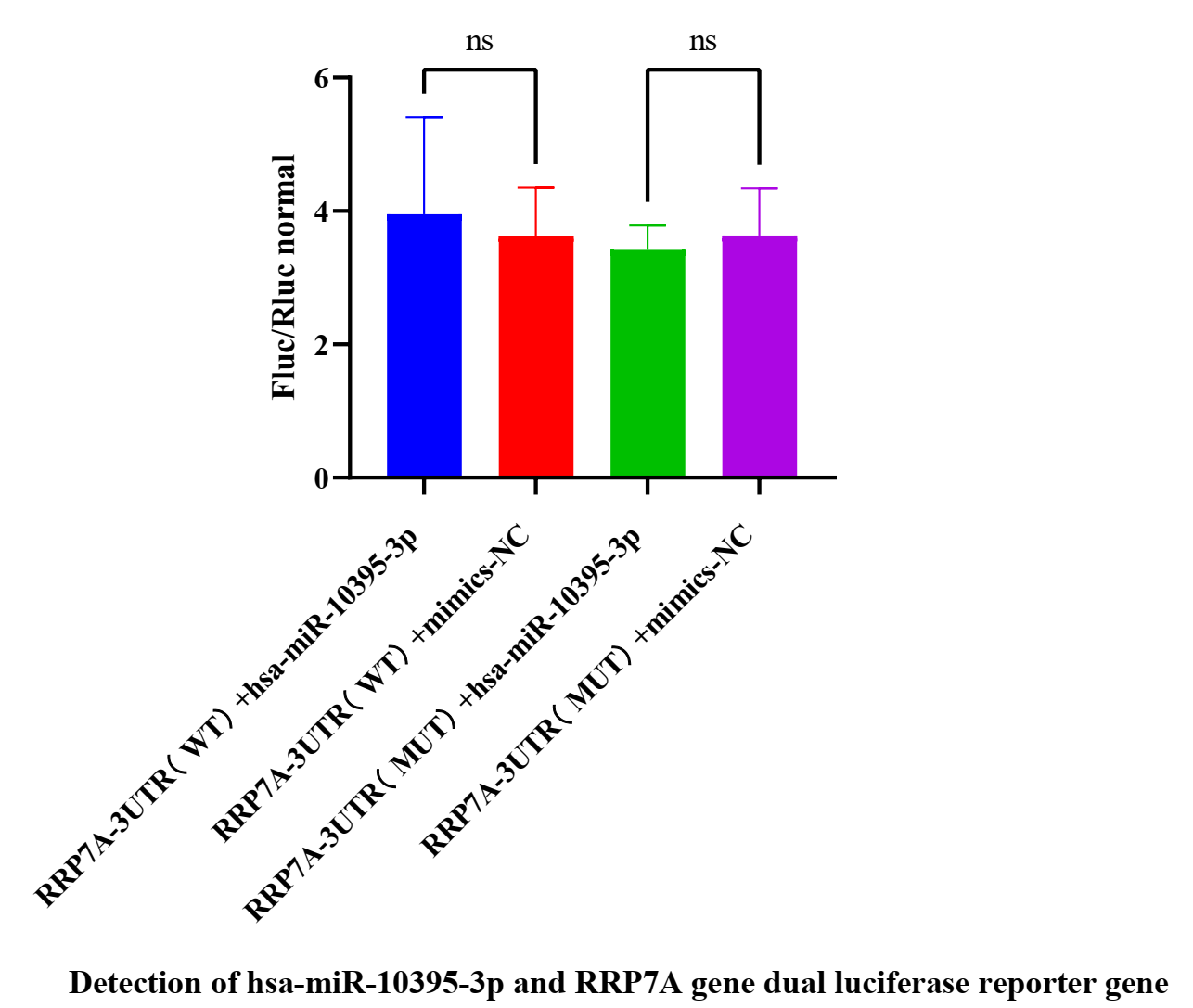 |

Note: * *p* < 0.05, ns *p* > 0.05. WT: wild type, MUT: mutant type.
